# Phenotypic diversity of genetic Creutzfeldt–Jakob disease: a histo-molecular-based classification

**DOI:** 10.1007/s00401-021-02350-y

**Published:** 2021-07-29

**Authors:** Simone Baiardi, Marcello Rossi, Angela Mammana, Brian S. Appleby, Marcelo A. Barria, Ignazio Calì, Pierluigi Gambetti, Ellen Gelpi, Armin Giese, Bernardino Ghetti, Jochen Herms, Anna Ladogana, Jacqueline Mikol, Suvankar Pal, Diane L. Ritchie, Viktoria Ruf, Otto Windl, Sabina Capellari, Piero Parchi

**Affiliations:** 1 IRCCS Istituto delle Scienze Neurologiche di Bologna, Ospedale Bellaria, Via Altura 1/8, 40139 Bologna, Italy; 2 Department of Experimental, Diagnostic and Specialty Medicine (DIMES), University of Bologna, Bologna, Italy; 3 Department of Pathology, School of Medicine, Case Western Reserve University, Cleveland, OH USA; 4 National Prion Disease Pathology Surveillance Center (NPDPSC), School of Medicine, Case Western Reserve University, Cleveland, OH USA; 5 National CJD Research & Surveillance Unit, Centre for Clinical Brain Sciences, Western General Hospital, University of Edinburgh, Edinburgh, UK; 6 Division of Neuropathology and Neurochemistry, Department of Neurology, Medical University of Vienna, Vienna, Austria; 7 Center for Neuropathology and Prion Research, Ludwig-Maximilians-Universität München, Munich, Germany; 8 Department of Pathology and Laboratory Medicine, School of Medicine, Indiana University, Indianapolis, IN USA; 9 Department of Neuroscience, Istituto Superiore di Sanità, Rome, Italy; 10 Service d’Etude des Prions et des Infections Atypiques, Université Paris-Saclay, CEA, Fontenay-aux-Roses, France; 11 Department of Biomedical and Neuromotor Sciences (DIBINEM), University of Bologna, Bologna, Italy

**Keywords:** Prion protein, *PRNP*, Fatal familial insomnia, FFI, Prion disease, CJD subtypes, Prion strains

## Abstract

The current classification of sporadic Creutzfeldt–Jakob disease (sCJD) includes six major clinicopathological subtypes defined by the physicochemical properties of the protease-resistant core of the pathologic prion protein (PrP^Sc^), defining two major PrP^Sc^ types (i.e., 1 and 2), and the methionine (M)/valine (V) polymorphic codon 129 of the prion protein gene (*PRNP*). How these sCJD subtypes relate to the well-documented phenotypic heterogeneity of genetic CJD (gCJD) is not fully understood. We analyzed molecular and phenotypic features in 208 individuals affected by gCJD, carrying 17 different mutations, and compared them with those of a large series of sCJD cases. We identified six major groups of gCJD based on the combination PrP^Sc^ type and codon 129 genotype on *PRNP* mutated allele, each showing distinctive histopathological characteristics, irrespectively of the *PRNP* associated mutation. Five gCJD groups, named M1, M2C, M2T, V1, and V2, largely reproduced those previously described in sCJD subtypes. The sixth group shared phenotypic traits with the V2 group and was only detected in patients carrying the E200K-129M haplotype in association with a PrP^Sc^ type of intermediate size (“i”) between type 1 and type 2. Additional mutation-specific effects involved the pattern of PrP deposition (e.g., a “thickened” synaptic pattern in E200K carriers, cerebellar “stripe-like linear granular deposits” in those with insertion mutations, and intraneuronal globular dots in E200K-V2 or -M”i”). A few isolated cases linked to rare *PRNP* haplotypes (e.g., T183A-129M), showed atypical phenotypic features, which prevented their classification into the six major groups. The phenotypic variability of gCJD is mostly consistent with that previously found in sCJD. As in sCJD, the codon 129 genotype and physicochemical properties of PrP^Sc^ significantly correlated with the phenotypic variability of gCJD. The most common mutations linked to CJD appear to have a variable and overall less significant effect on the disease phenotype, but they significantly influence disease susceptibility often in a strain-specific manner. The criteria currently used for sCJD subtypes can be expanded and adapted to gCJD to provide an updated classification of the disease with a molecular basis.

**Supplementary Information:**

The online version contains supplementary material available at 10.1007/s00401-021-02350-y.

## Introduction

Prion diseases are invariably fatal, largely transmissible, neurodegenerative disorders that affect humans and other mammals. The central pathogenic event in prion disease is the accumulation of a misfolded, prone-to-aggregate isoform of the cellular prion protein (PrP^C^), named scrapie isoform (PrP^Sc^). The transition to PrP^Sc^ involves the refolding of PrP^C^ into high‐β‐sheet conformers that assemble into amyloid fibrils. Once formed, PrP^Sc^ replicates itself by a seeded-conversion mechanism in which PrP^Sc^ multimers bind to PrP^C^ and mediate its conversion into PrP^Sc^ [87].

Prion diseases typically manifest a wide range of phenotypic variability, depending on genetic factors, namely, the primary sequence of the prion protein gene (*PRNP*), and PrP^Sc^ intrinsic properties likely related to the conformational heterogeneity of PrP^Sc^ multimers. PrP^Sc^ structural diversity often generates biological variants, termed strains, that can be serially propagated upon experimental or natural transmission independently from the host genotype [7, 11, 78, 98]. Prion strains can be distinguished after transmission to syngeneic hosts by the regional lesion profile, morphology of PrP^Sc^ deposits, and biochemical properties of PrP^Sc^ reflecting the distinct quaternary structure of PrP^Sc^ aggregates [2, 5]. Following their initial discovery and characterization through the experimental transmission of scrapie isolates [11], prion strains have also been associated with human prion diseases' phenotypes [8, 46, 51, 71, 84, 92, 101]. The current classification of sporadic Creutzfeldt–Jakob disease (sCJD), the most common human prion disease, recognizes six major phenotypic subtypes with distinctive clinicopathological features, broadly correlating at the molecular level with the genotype at the polymorphic codon 129 (methionine, M; or valine, V) in the prion protein gene (*PRNP*) and the size of the protease-resistant PrP^Sc^ core (type 1 migrating at 21 kDa and type 2 at 19 kDa), which is a prion strain marker in individuals with the same codon 129 genotype [79, 92].

In contrast to animal prion diseases, the human prion disease includes genetic forms linked to mutations in the *PRNP* gene encoding for PrP^C^. The genetic prion disease accounts for up to 15% of all cases and manifests an autosomal dominant inheritance pattern with variable penetrance. *PRNP* mutations are associated with a wide range of clinicopathological entities, encompassing CJD, Gerstmann–Sträussler–Scheinker disease (GSS), and fatal familial insomnia (FFI) [26, 29, 56]. The existence of *PRNP* mutations that segregate with familial prion diseases [38, 39, 64, 75] raises the critical question of these mutations' pathogenic role, including their effect on disease phenotype and the generation of strain-specific properties. The issue is particularly relevant for the mutations linked to CJD and FFI, for which sporadic and genetic disease forms are firmly established. Notably, a significant number of patients with CJD carrying a mutation lack a positive family history, while only a subgroup of *PRNP* mutations show high or full penetrance [52, 66].

In genetic prion disorders, the *PRNP* primary sequence was initially thought to be the main determinant of phenotypical heterogeneity [10]. However, increasing evidence indicates that genetic phenotypes largely reproduce the spectrum of the sporadic ones. In line with this view, initial experimental studies demonstrated that the transmission of genetic CJD (gCJD) to mice, monkeys, and bank voles induces disease phenotypes indistinguishable from those determined by sCJD inocula [4, 51, 58, 61, 71, 84, 96]. Notably, preliminary data support the hypothesis that distinct phenotypes in genetic prion diseases also behave as specific strains and that the same strain can affect patients carrying different *PRNP* mutations [71, 84, 98]. Despite the above, a systematic analysis of molecular and clinicopathological features across the spectrum of *PRNP* variants and their associated disorders is lacking. Indeed, given the low disease prevalence, most prior studies have focused on phenotypes related to a single *PRNP* mutation and a limited number of participants. To contribute to the full understanding of these issues and reach a better classification of genetic prion diseases, we performed a comprehensive study of molecular and phenotypic features in an extensive patient cohort, and compared them with their sporadic counterparts. Since the GSS phenotype does not have an established sporadic phenotype, we focused on patients with CJD and fatal insomnia (FI).

## Materials and methods

### Selection of patients

We studied 208 *PRNP* mutation carriers with a neuropathologically confirmed diagnosis of either CJD or FFI.

One hundred and fifty-one patients died in Europe (Italy, 98; Germany, 36; UK, 10; France, 6, and Austria, 1) and 57 in the USA (for a detailed list of providing Centers see Supplementary methods, online resource). Comprehensive data for 20 subjects and partial information of clinical and/or pathological and/or molecular genetic features for additional 17 patients have been previously reported [9, 10, 12, 16, 19, 24, 27, 31–33, 35, 40, 55, 57, 60, 65, 68, 69, 76, 77, 89–91, 95, 99]. Inclusion criteria were limited to the availability of CNS frozen tissue for PrP^Sc^ typing and determination of the *PRNP* haplotype (i.e., mutation plus the genotype at codon 129 in *cis*). As the only exception, given the rarity of the molecular combination, two cases that lacked frozen tissue but belonged to well-characterized families carrying the D178N-129V haplotype were also included [12, 60].

Finally, to compare the biochemical and pathological heterogeneity between genetic and sporadic forms of CJD and FI, we used a previously published cohort of 225 individuals with sCJD and 6 with sporadic FI [1, 80, 82].

### Histological examination

Semiquantitative evaluation of spongiform change and astrogliosis was carried out in 193 brains by comparing hematoxylin and eosin stained sections from the affected participants and the sporadic CJD and FI subjects. Spongiform change was scored on a 0–4 scale (not detectable, mild, moderate, severe, and status spongiosus), whereas astrogliosis was scored on a 0–3 scale (not detectable, mild, moderate, and severe). A lesion profile for each patient was obtained by averaging the two scores. Only in the FI group, we analyzed the extent of spongiform change and astrogliosis separately. At least nine anatomical regions were always analyzed, including frontal, temporal and occipital neocortices, CA1 sector of hippocampus, entorhinal cortex, striatum (caudate nucleus and putamen), midbrain (substantia nigra and periaqueductal gray), medial thalamus, and cerebellum (vermis and hemisphere).

### Immunohistochemistry

Paraffin sections from formalin-fixed tissue blocks of frontal (*n* = 175) and occipital cortices (*n* = 176), thalamus (*n* = 146) and cerebellum (*n* = 186) obtained from 190 brains, were immunolabelled using the monoclonal antibodies 3F4, 12F10 or L42 (German cases), as previously described [82, 85, 100]. The main pattern of PrP deposition, reflecting the morphology of PrP aggregates was analyzed in each brain region, and compared with those previously described in sCJD (e.g., “synaptic”; coarse/perivacuolar; plaque-like) [79]. In a subgroup of cases showing the plaque-like pattern, we also performed an analysis of the aggregate size with the Aperio Imagescope software using 10 sections (363.5 × 241.8 μm, 5 from cerebellum and 5 from the neocortex) for each case.

### Molecular genetic analysis

*PRNP* (RefSeq NM_0003111) open reading frame (ORF) was analyzed as previously described [16, 79]. Briefly, genomic DNA was used to amplify the *PRNP* coding region in the polymerase chain reaction with the primers Forward 5´-GCAGTCATTATGGCGAACCTTGGCTG-3´ and Reverse 5´-GTACTGAGGATCCTCCTCATCCCACTATCAGGAAGA-3´. Mutation and codon 129 genotype were determined by direct sequencing of the *PRNP* ORF using the BigDye Terminator 3.1 Cycle Sequencing Kit according to manufacturer’s instructions and the Applied Biosystems ABI3500 Dx Genetic Analyzer sequencer (Thermo Fisher Scientific, Waltham, MA). To ascertain the codon 129 genotype in the mutated allele in MV heterozygotes, the amplified ORF was digested with a mutation-specific restriction enzyme, and then re-amplified with the same primers. The codon 129 genotype of the allele bearing the mutation was then determined by direct sequencing. For insertion/deletion mutations, the genotype at codon 129 in *cis* with the mutation was determined as described [3].

### Protein studies

#### PrP^Sc^ typing

Immunoblot analysis of PrP^Sc^ was carried out as previously described [83]. One or multiple samples from different brain regions, including the frontal cortex (*n* = 202), temporal cortex (*n* = 158), occipital cortex (*n* = 155), putamen (*n* = 149), medial thalamus (*n* = 152), and cerebellum (*n* = 165) were examined in all but two subjects with the D178N-129V *PRNP* haplotype (see also patient selection). To evaluate in depth the extent of co-occurrence of mixed PrP^Sc^ types, we performed immunoblots in samples from all six regions in 142 cases. Sample preparation and immunoblot analysis of PrP^Sc^ was carried out as previously described using 7 or 15 cm long separating gels [73, 83, 91].

#### PK titration curves

Serial PK digestion of total homogenates (THs) from the frontal cortex was performed as described [91]. Briefly, THs were adjusted to a total protein concentration of 6 mg/ml, and the applied PK activity ranged from 2 to 256 U/ml. ED_50_ expresses the PK concentration needed to digest 50% of PrP^Sc^.

#### Thermo-solubilization assay (TSA)

The analysis of thermostability of PrP^Sc^ aggregates was performed as described [22]. Briefly, THs were digested with 8 U/ml PK for 1 h at 37 °C with mild shaking (300 rpm). After PK inactivation by PMSF (3.6 mM final concentration), aliquots were mixed with an equal volume of loading buffer (final concentration 1.5% SDS, 2% β-mercaptoethanol, 5% glycerol, 1 mM EDTA, 31.2 mM Tris, pH 6.8) and heated to temperatures ranging from 25 °C to 95 °C (∆*T* = 10 °C) for 6 min before immunoblot analysis. T_50_ expresses the temperature needed to solubilize 50% of PrP^Sc^.

PK titration curves and TSA analyses were performed in 50 gCJD and 47 sCJD cases, which are described in detail in Supplementary Table 1, online resource.

### Clinical data and diagnostic investigations

Clinical data including at least those obtained from one neurological examination were available in 192 (179 gCJD and 13 FFI) patients. Disease duration was calculated from the time of presentation of neurological signs to death (i.e., prodromal nonspecific symptoms were not considered). We also reviewed the clinical charts for the following: symptoms at onset and during disease evolution, results of electroencephalographic recordings (EEG), cerebral magnetic resonance imaging (MRI) studies, and cerebrospinal fluid analyses, including results of proteins 14-3-3, and total-tau (t-tau), and of the prion real-time quaking-induced conversion (RT-QuIC) assay (see also Supplementary Materials, online resource).

## Results

### Molecular genetic analysis

All participants carried a single heterozygous *PRNP* mutation, except for one who was homozygous for the R208H mutation. The distribution in the cohort of *PRNP* haplotypes determined by the mutation and the codon 129 polymorphism in both mutated and normal alleles is summarized in Table 1.

**Table 1:** Distribution of PrP^Sc^ type according to *PRNP* haplotype

Codon 129	129M								129V					
*PRNP* mutation	D178N	E196A/K	E200K	R208H	V210I	3/4-OPRI	5/6-OPRI	Others A^§^	D178N	T188R	E200K	5/6-OPRI	Others B^¶^	Total (%)
*n*	13	6	70	7	51	6	7	9	11	6	5	12	3	206
129 MV (%)	6 (46.2)	1 (16.7)	16 (22.9)	0	11 (21.6)	0	3 (42.9)	2 (22.2)	5 (45.5)	2 (33.3)	2 (40.0)	3 (25.0)	0	51 (24.8)
Type 1 (%)	0	5 (83.3)	58 (82.9)	7 (100)	47 (92.2)	6 (100)	4 (57.1)	6 (66.7)	11^a^ (100)	5^a^ (83.3)	0	0	0	149 (72.3)
Type 1 + 2 (%)	0	1 (16.7)	0	0	4 (7.8)	0	2 (28.6)	1 (11.1)	0	1 (16.7)	1 (20.0)	3 (25.0)	0	13 (6.3)
Type 2 (%)	13 (100)	0	4 (5.7)	0	0	0	1 (14.3)	2 (22.2)	0	0	4 (80.0)	9^b^ (75.0)	3 (100)	36 (17.5)
Type “i” (%)	0	0	8 (11.4)	0	0	0	0	0	0	0	0	0	0	8 (3.9)

^§^Others A includes: V203I PrP^Sc^ type 1 (*n* = 2) and type 1 + 2 (*n* = 1), T188K, type 1 (*n* = 1), D211Q, type 1 (*n* = 2), R148H, type 1 (*n* = 1), T183A, type 2 (*n* = 2)

^¶^Others B includes: T188A PrP^Sc^ type 2 (*n* = 1), E196K, type 2 (*n* = 1), R208H, type 2 (*n* = 1)

3/4-OPRI-129M includes: 3-OPRI PrP^Sc^ type 1 (*n* = 1), and 4-OPRI, type 1 (*n* = 5). 5/6-OPRI-129M includes: 5-OPRI PrP^Sc^ type 1 (*n* = 1), type 1 + 2 (*n* = 2) and type 2 (*n* = 1), and 6-OPRI type 1 (*n* = 2). 5/6-OPRI-129V includes: 5-OPRI PrP^Sc^ type 2 + ”i” (n = 2), type 2 (*n* = 6), and “atypical” type 1 + 2 (*n* = 2), and 6-OPRI type 2 + ”i” (*n* = 1) and “atypical” type 1 + 2 (*n* = 1)

^a^In most cases the unglycosylated band migrated as a doublet of fragments, in variable relative proportion, including the 21 kDa (type 1) fragment and a second slightly faster-migrating fragment

^b^Includes 3 cases characterized by an unglycosylated PrP^Sc^ isoform migrating as a doublet (type 2 + “i”)

PrP^Sc^ typing was not performed in two patients carrying the D178N-129V haplotype (129 MV) (see methods)

### PrP^Sc^ typing

**Fig. 1:**
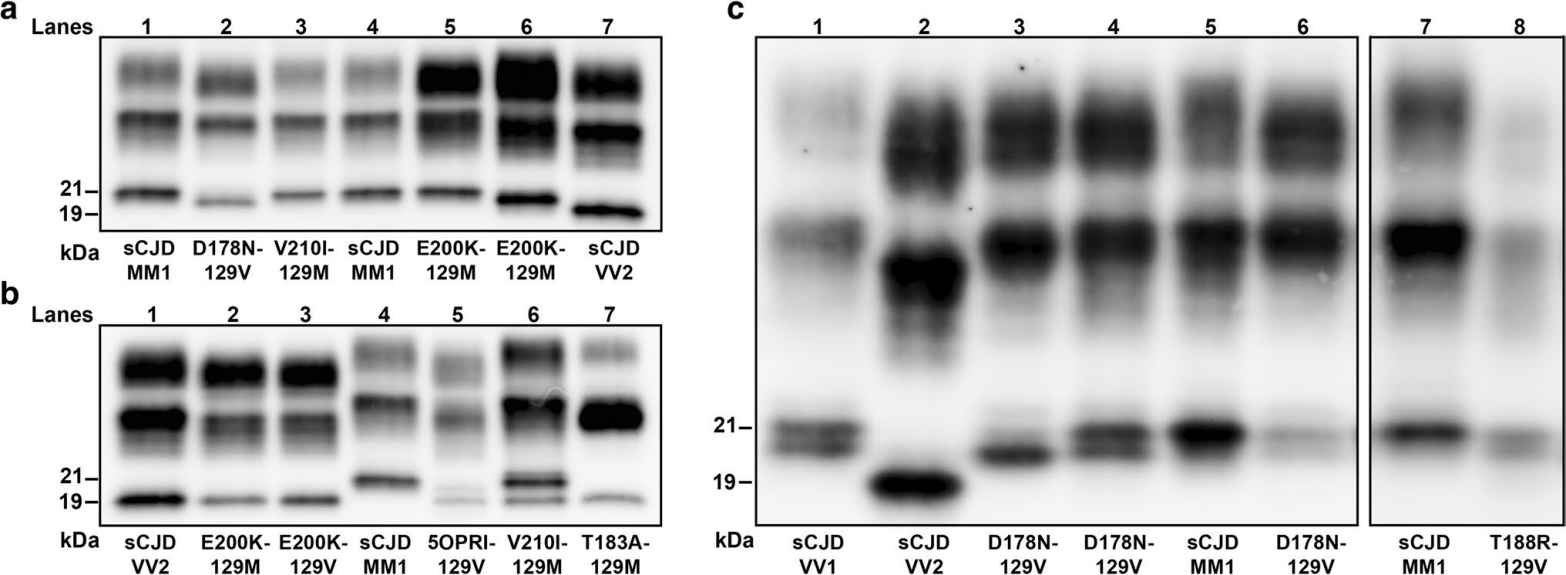
Immunoblot profile of PrP^Sc^ in genetic CJD. **a** Genetic CJD haplotypes associated with PrP^Sc^ type 1 (lanes 3, 5) and “i” (lane 6). As in sCJD MM1, monoglycosylated PrP^Sc^ was predominant in the V210I mutation, while the diglycosylated was the most represented isoform in patients carrying the E200K variant. D178N-129V carriers showed an equal mix of mono- and diglycosylated isoforms, whereas the unglycosylated band was underrepresented. **b** Genetic CJD haplotypes associated with PrP^Sc^ type 2 (lanes 2, 3, 5 and 7) and 1 + 2 (lane 6). The unglycosylated isoform in a 5-OPRI-129V case with heterozygosity (MV) at codon 129 (lane 5) is characterized by a doublet migrating at 19 and 20 kDa (PrP^Sc^ type 2 + “i”). The T183A mutation showed a profile characterized by a marked under-representation of the diglycosylated isoform as compared with the monoglycosylated band. **c** Immunoblot profile of D178N-129V cases. The unglycosylated isoform is represented by doublet with variable dominance of either the band migrating at 21kDa (PrP^Sc^ type 1) or the one migrating slightly faster. A similar pattern of migration was observed in sCJD VV1 (lane 1). a2 and c3 lanes showed the same case. Samples were resolved in 7 (**a, b**) and 15 cm (**c**) long gels and probed with the primary antibody 3F4

In most cases, the immunoblot profile of proteinase-K (PK)-resistant PrP^Sc^ reproduced the type 1 (~ 21 kDa) and type 2 (~ 19 kDa) profiles previously described in sCJD (Fig. 1) [78]. However, eight participants carrying the E200K-129M haplotype and V at codon 129 in the wild-type allele showed a type “i” (= intermediate) profile characterized by a 20 kDa unglycosylated PrP^Sc^ core (Fig. 1a). Moreover, as previously observed in sCJD MV2K [72, 82], three MV heterozygotes carrying an insertional mutation of 5 or 6 octapeptide repeats in *cis* with 129V (5/6-OPRI-129V) showed a doublet of unglycosylated fragments migrating at 19 kDa (type 2) and 20 kDa (type “i”) (Fig. 1b). Similarly, participants carrying the D178N-129V and T188R-129V haplotypes showed an unglycosylated PrP^Sc^ band comprising a doublet of fragments, in variable relative proportion, including the 21 kDa (type 1) peptide and a second slightly faster-migrating fragment (Fig. 1c). Finally, a subgroup of participants carrying 5/6-OPRI-129V also showed a PrP^Sc^ type 1 profile comprised of a doublet of fragments, including a second fragment migrating slightly slower than the typical 21 kDa (type 1) associated band (Supplementary Fig. 1, online resource).

PrP^Sc^ type 1 and type 2 were significantly associated with both codon 129 genotype and mutation type (Table 1). Similarly to sCJD (Supplementary Table 2, online resource), the individuals carrying the prevalent mutations, i.e., E200K, V210I, and OPRI, generally showed PrP^Sc^ type 1 when coupled with 129M, and type 2 (or type 2 + “i”) when associated with 129V. Exceptions included the eight cases carrying E200K-129M combined with PrP^Sc^ type “i”, and five individuals with E200K-129M or 5-OPRI-129M in association with PrP^Sc^ type 2. In contrast, a few rare mutations (T183A, T188R), and the prevalent D178N variant showed either an exclusive association with PrP^Sc^ type 2 when in *cis* with M (T183A) or an almost invariable association with PrP^Sc^ type 1 when in *cis* with V (T188R) or both associations (D178N) (Table 1).

Regional immunoblot analysis showed PrP^Sc^ types 1 + 2 co-occurrence in a minority (6.3% overall) of participants carrying the following haplotypes: V210I-129M, 5/6-OPRI-129M, V203I-129M, E196K-129M, E200K-129V, T188R-129V, and 5/6-OPRI-129V (Table 1). A sub-analysis limited to the cases with at least six brain regions available for immunoblotting demonstrated only a slight increase in the prevalence of PrP^Sc^ types co-occurrence (7.0%) (Supplementary Table 3, online resource).

As previously shown [21, 32, 37, 81], the relative proportion of the three bands that correspond to the di-, mono-, and unglycosylated PrP^Sc^ differed significantly between participants carrying different *PRNP* mutations (Supplementary Table 4, online resource). As in sCJD, monoglycosylated PrP^Sc^ was generally predominant. However, the diglycosylated form was the most represented isoform in those carrying E200K and D178N-129M. Moreover, participants carrying the D178N-129V haplotype showed an equal mix of mono- and diglycosylated isoforms, whereas the unglycosylated band was underrepresented. Finally, the T183A and the 5/6-OPRI-129V subgroup with PrP^Sc^ types 1 and 2 co-occurrence had peculiar immunoblot profiles characterized by a marked under-representation of the diglycosylated isoform (Fig. 1, Supplementary Figs. 1 and 2, online resource).

### Histotype classification of gCJD

Given the strong molecular phenotypic correlation, we classified the study cohort into five major groups based on PrP^Sc^ type and codon 129 genotype of the *PRNP* mutated allele. Thereafter, we analyzed the effect of *PRNP* mutations within each of the five groups, namely 129M-type 1, 129V-type 2, 129M-type 2, 129V-type 1, and 129M-type “i.” Given the atypical PrP^Sc^ features associated with T183A-129M cases and a subgroup of participants carrying 5 or 6 OPRI and 129V, we characterized these cases separately.

#### Group 129M-type 1

**Fig. 2:**
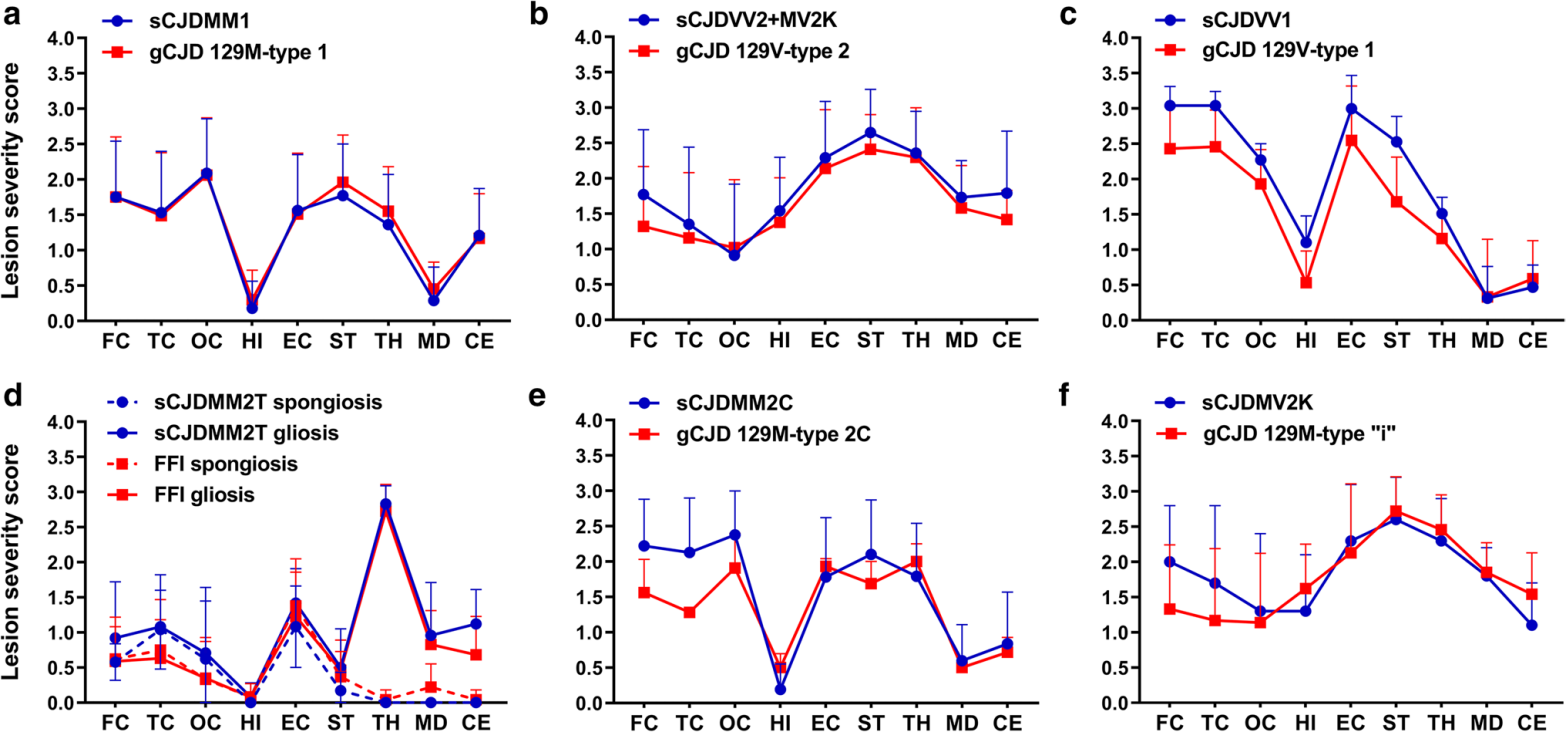
Comparison of lesion profiles between the different genetic CJD groups classified according to the *PRNP* haplotype/PrP^Sc^ type combination and the corresponding sCJD subtypes. The following anatomical regions were analyzed: frontal (FC), temporal (TC), and occipital (OC) neocortices, hippocampus (HI) (CA 1 region), entorhinal cortex (EC), neostriatum (ST) (nuclei caudatus and putamen), thalamus (TH) (mediodorsal nucleus), midbrain (MD) (substantia nigra and periaqueductal gray), and cerebellum (CE). Spongiform change was scored on a 0–4 scale (not detectable, mild, moderate, severe, and status spongiosus), and gliosis on a 0–3 scale (not detectable, mild, moderate, and severe). Lesion profiles were obtained by averaging the two scores for each brain region examined. Spongiform change and gliosis are shown separately in FFI and sCJD MM2T. Data are expressed as mean ± SD values

**Fig. 3:**
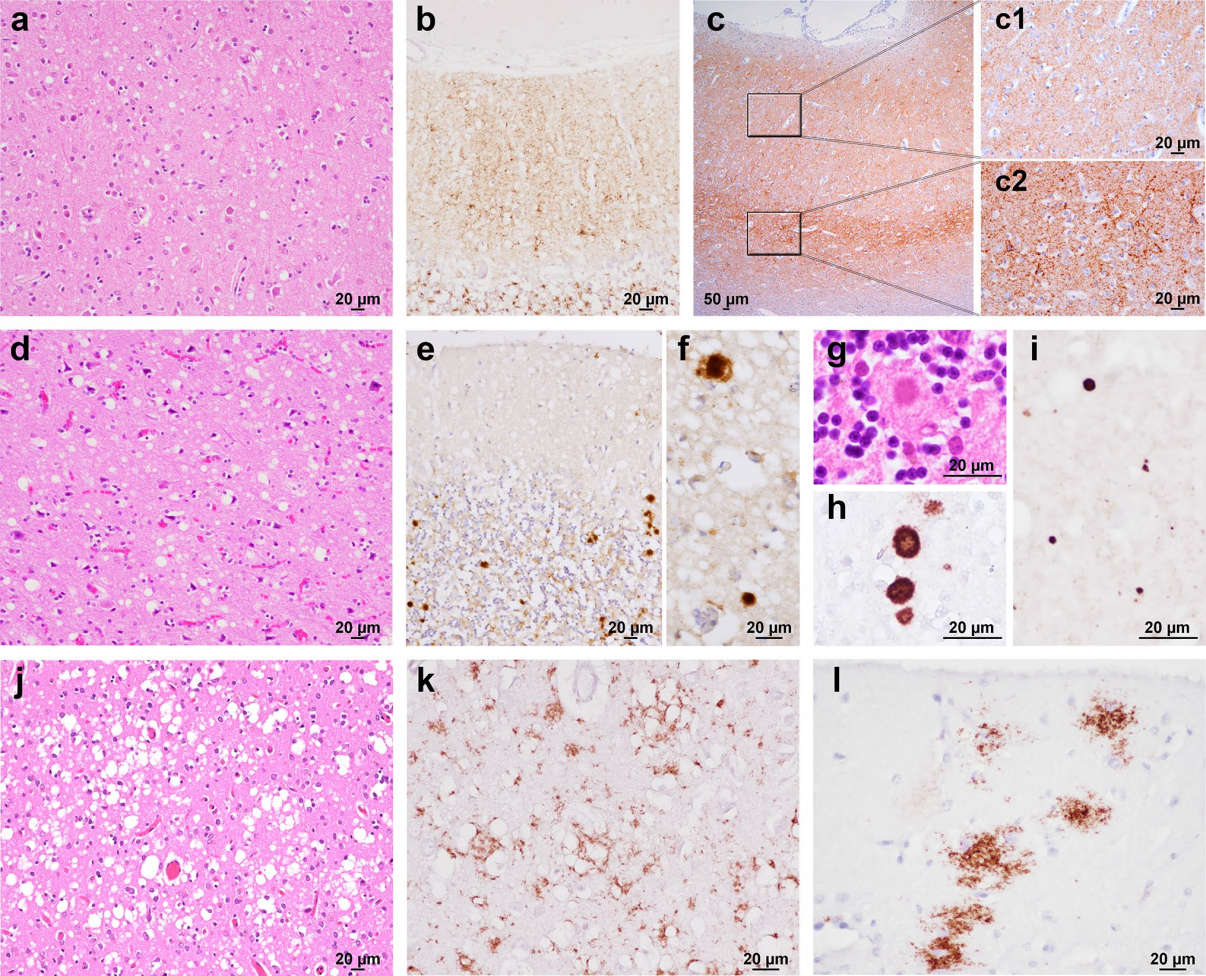
Main histopathological features of genetic CJD phenotypes. **a, b** Spongiform change characterized by small, non-confluent vacuoles (**a**, frontal cortex, E200K-129M), and synaptic type of PrP deposition (**b**, cerebellum, V210I-129M) in subjects of the 129M-type 1 group. **c** Prominent PrP immunoreactivity in the deep cortical layers, i.e., laminar distribution, in a subject carrying E200K-129V (frontal cortex; details at higher magnification in **c1** and **c2**). **d** Spongiform change with intermediate size vacuoles in a D178N-129V carrier (temporal cortex). **e–h** Plaque-like PrP deposition in the cerebellar granular layer (**e**) and in the frontal cortex (**f**); kuru-type plaques (**g, h**) in the cerebellar granular layer of a codon 129 heterozygous patient with 5-OPRI-129V. **i** Mini plaque-like PrP deposits in the subiculum of a D178N-129V carrier. **j, k** Spongiform change with large and confluent vacuoles (**j**) and coarse PrP deposits with perivacuolar distribution (**k**) in the occipital cortex of an individual of the E200K-type 2C group. **l** Patchy PrP deposits in the molecular layer of a case of the E200K-type 2C group. Haematoxylin–eosin staining (**a, d, g, j**), and immunohistochemistry for PrP with mAb 3F4 (**b, e, f, h, i, k, l**), and 12F10 (**c**)

**Fig. 4:**
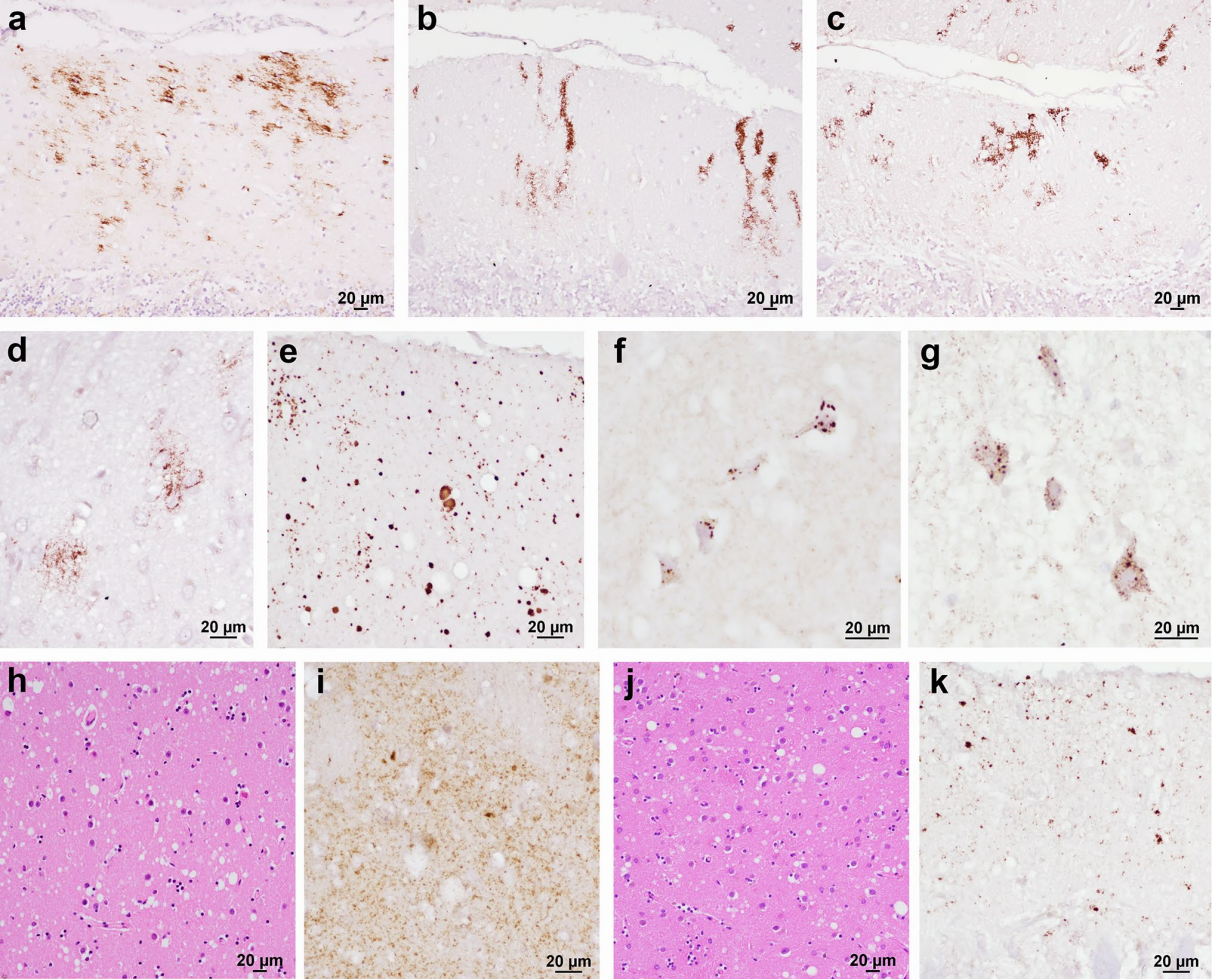
Mutation-specific histopathological variations across the spectrum of gCJD phenotypes.** a** Modified, “thickened” synaptic pattern of PrP deposition in the molecular layer of cerebellum in a patient carrying the E200K-129M haplotype. **b, c** Granular PrP deposits with long-thin (**b**) and short-thick (**c**) stripe-like appearance distributed perpendicularly to the surface in the molecular layer of cerebellum in 6- and 4-OPRI-129M carriers, respectively. **d** Round patches of fine, granular PrP deposits in the deep frontal cortex of a 4-OPRI-129M case. **e** Multiple, small PrP-amyloid plaques in the cerebellar molecular layer in a E211Q-129M carrier. **f, g** Multiple, intraneuronal globular PrP deposits distributed in the perikaryon in a E200K-129V individual with PrP^Sc^ type 2 (**f**, deep layers of frontal cortex) and E200K-129M with PrP^Sc^ type “i” (**g**, pons). **h–k** Spongiform change characterized by small and intermediate, non-confluent vacuoles in the caudate nucleus, and granular/mini plaque-like PrP deposits in the striatum and molecular layer of cerebellum of patients with T183A-129M (**h, i**) and atypical 6-OPRI-129V (**j, k**), respectively. Immunohistochemistry for PrP with mAb 3F4 (**a–g, i, k**), and haematoxylin–eosin staining (**h, j**)

The 129M-PrP^Sc^ type 1 combination was the most frequent (*n* = 141) and included several *PRNP* mutations (Table 1). The type of spongiform change, lesion profile, and PrP deposition pattern were highly consistent among cases. However, some mutation-specific effects were evident, particularly in the morphology of PrP deposits. As in sCJD MM(V)1 [85], the spongiform change, characterized by small, non-confluent vacuoles, mainly affected the cerebral cortices, neostriatum, thalamus, and cerebellum, whereas the hippocampus and midbrain were relatively spared (Figs. 2a and 3a). Moreover, like in sCJD MM(V)1, immunohistochemistry demonstrated a synaptic pattern of PrP deposition in the cerebellum and the cerebral cortex (Table 2, Fig. 3b, Supplementary Table 5, online resource). Mutation-specific phenotypic variations involved the E200K, E196K/A, and OPRI carriers (Fig. 4). Participants with E200K and E196K/A (47.8% and 66.7%, respectively) showed a modified synaptic pattern (hereafter designated as “thickened” synaptic), characterized by focal, patchy PrP aggregates in the molecular layer of the cerebellum and, to a lesser extent, in the cerebral cortices (Fig. 4a). Moreover, 10 out of 12 (83.3%) OPRI cases displayed small granular deposits with a striking linear distribution in the cerebellum molecular layer (Fig. 4b, c). These stripes extended perpendicularly from the molecular layer's surface to the Purkinje cell layer in 5/6-OPRI participants, while they were shorter and thicker in the carriers of 3 or 4 OPRI (3/4-OPRI). Of the two cases not displaying stripes, one had very severe, panencephalopathic changes and showed extensive synaptic PrP deposition in the cerebellar molecular layer and plaque-like deposits in the granular layer. The other one showed fine PrP deposits in the molecular layer, sometimes assuming a striking linear orientation perpendicular to the surface. Moreover, there were clusters ofgranular PrP deposits in round patches, often associated with non-confluent spongiform changes, in the deeper cortical layers layers (IV–V) of 8 out of 12 (66.7%) OPRI cases (Fig. 4d). Finally, one of the two participants (50%) carrying E211Q-129M showed numerous small, sometimes confluent PrP-amyloid plaques in the cerebellar molecular layer (Fig. 4e).

**Table 2:** Pattern of PrP^Sc^ deposition in gCJD and FFI groups

gCJD groups	*n*	Cerebellar or cortical synaptic^§,^^¶^	Cortical coarse/perivacuolar	Cerebellar (G.L.) or thalamic plaque-like	Cerebellar (M.L.) and cortical	Cerebellar kuru plaques	Intra-neuronal globular
and *PRNP* mutations					mini plaque-like		
		*n* (%)	*n* (%)	*n* (%)	*n* (%)	*n* (%)	*n* (%)
**129M-type 1**	**127**	**127 (100)**	**39 (30.7)**	**3 (2.3)**^¥^	**0 (0.0)**	**0 (0.0)**	**0 (0.0)**
V210I	50	50 (100)	24 (48.0)	1 (2.0)	–	–	–
E200K	46	46 (100)	11 (23.9)	–	–	–	–
3 to 6-OPRI	12	12 (100)	2 (16.7)	1 (8.3)	–	–	–
R208H	6	6 (100)	1 (16.7)	1 (16.7)	–	–	–
E196A/K	6	6 (100)	–	–	–	–	–
Others A^$^	7	7 (100)	1 (14.3)^#^	–	–	–	–
**129V-type 2**	**17**	**14 (82.3)**	**0 (0.0)**	**17 (100)**	**0 (0.0)**	**4 (23.5)**	**5 (29.4)**
5/6-OPRI	9	7 (77.8)	–	9 (100)	–	3 (33.3)	–
E200K	5	5 (100)	–	5 (100)	–	–	5 (100)
Others B^&^	3	2 (66.7)	–	3 (100)	–	1 (33.3)*	–
**129V-type 1**	**15**	**0 (0.0)**	**0 (0.0)**	**0 (0.0)**	**15 (100)**	**0 (0.0)**	**0 (0.0)**
D178N	8	–	–	–	8 (100)	–	–
T188R	7	–	–	–	7 (100)	–	–
**129M-type 2**	**18**	**9 (50.0)**	**5 (27.8)**	**0 (0.0)**	**0 (0.0)**	**0 (0.0)**	**0 (0.0)**
D178N	13	5 (38.5)	–	–	–	–	–
E200K	4	4 (100)	4 (100)	–	–	–	–
5-OPRI	1	–	1 (100)	–	–	–	–
**129M-type “i”**	**8**	**8 (100)**	**0 (0.0)**	**0 (0.0)**	**0 (0.0)**	**0 (0.0)**	**8 (100)**
E200K	8	8 (100)	**–**	^**−**^	**–**	**–**	8 (100)
Atypical	5	2 (40.0)	0 (0.0)	0 (0.0)	5 (100)	0 (0.0)	0 (0.0)
T183A^a^	2	1 (50.0)	–	–	2 (100)	–	–
5/6-OPRI^b^	3	1 (33.3)	–	–	3 (100)	–	–

Bold values indicate the results obtained in the six histo-molecular gCJD groups irrespective of the mutations

*G*.*L*. granular layer, *M*.*L*. molecular layer

^§^A modified, “thickened” synaptic pattern of PrP deposition was evident in: 22 (47.8%) E200K 129M-type 1, 4 (66.7%) E196A/K 129M-type 1, 2 (40.0%) E200K 129V-type 2, 4 (100%) E200K 129M-type 2, and 3 (37.5%) E200K 129M-type “i”

^¶^A modified, synaptic pattern with cerebellar “stripes” in the M.L. was evident in: 10 (83.3%) OPRI 129M-type 1, 9 (100%) OPRI 129V-type 2, and 1 (33.3%) OPRI 129V- “atypical” type 1 + 2

^$^Others A included: V203I, *n* = 3; T188K, *n* = 1; R148H, *n* = 1; D211Q, *n* = 2

^&^Others B included R208H, *n* = 1; E196K, *n* = 1; T188A, *n* = 1

^#^Patient carrying the V203I variant

^¥^The 3 cases showing plaque-like PrP deposits had a disease duration significantly longer compared with the others of the 129M-type 1 group (V210I, 18 months; 5-OPRI, 121 months; R208H, 11 months) and showed a severe panencephalopathic neuropathologic phenotype

^a^129M-type 2

^b^129V-type 1 + 2

^*^R208H-129V

#### Group 129V-type 2

In contrast to 129M-type 1, the lesion profile of 129V-type 2 cases showed a predominant involvement of the limbic cortices, deep brain nuclei, and midbrain. The cerebellum was variably affected, while pathological changes in the neocortices were less severe than those in the subcortical structures, particularly in the occipital lobe (Fig. 2b). Notably, as in sCJD VV2 and MV2K [85], the cortical spongiform change featured non-confluent vacuoles of intermediate size and often showed a laminar distribution with a predominant involvement of deeper layers.

We detected PrP-immunopositive amyloid plaques of the kuru type in the cerebellum of all the codon 129 MV patients carrying OPRI mutations (5-OPRI *n* = 2, 6-OPRI *n* = 1) and in the VV participant with R208H substitution (Fig. 3g, h). In contrast, kuru-type plaques were absent in codon 129 MV patients carrying the E200K mutation. Immunohistochemistry also revealed plaque-like PrP deposits in the cerebellar granular layer (Fig. 3e), thalamus, and, to a lesser extent, deep cortical layers close to the gray–white matter junction (Fig. 3f). The plaque-like pattern of PrP deposition was generally more pronounced in 5/6-OPRI than in E200K cases. Finally, there was a synaptic pattern of staining in 82.3% of cases (Table 2). Neocortical synaptic deposits often co-distributed with the laminar spongiform change in the deeper layers (Fig. 3c). Notably, all E200K-129V brains showed peculiar intraneuronal globular deposits, mainly involving the perikaryon, which was most evident in the brainstem nuclei, deeper layers of the cerebral cortex, and cerebellar dentate nucleus (Fig. 4f). Finally, similar to the OPRI mutations of the 129M-type 1 group, all the 5/6-OPRI showed linear stripes of PrP deposits in the cerebellar molecular layer.

#### Group 129V-type 1

Patients belonging to this group carried either the D178N or T188R variant. As in sCJD VV1 [85], 129V-type 1 participants showed a predominant cortico-striatal pathology and a relative sparing of the cerebellum and brainstem. Moreover, the hippocampus (CA1 and dentate gyrus) and, particularly, the entorhinal cortex were consistently affected (Fig. 2c). There were no significant differences between codon 129 VV and MV carriers (Supplementary Fig. 3, online resource). As in 129V-type 2 cases, spongiform change consisted of mostly non-confluent vacuoles of intermediate size (Fig. 3d). In all the cases, immunohistochemistry revealed small, round PrP aggregates in the molecular layer of the cerebellum and neocortices, where they often co-distributed with the most severe spongiform change in the deeper layers (Fig. 3i). PrP deposits in the neocortices were more abundant and significantly smaller than those detected in 129V-type 2 brains (Supplementary Fig. 4, online resource); hence, the eponym “mini plaque-like” was used to distinguish them from the plaque-like pattern seen in 129V-type 2 brains.

The D178N mutation carriers showed a homogeneous pattern of PrP deposition between the cortex and cerebellum. In contrast, T188R participants displayed larger and more pronounced PrP deposits in the cerebellum than in the cerebral cortex, and slightly more severe lesions in the cerebellum compared to the D178N carriers (Supplementary Figs. 4 and 5). Finally, PrP mini plaque-like aggregates were more abundant and showed a larger size than in sCJD VV1 (Supplementary Table 4, online resource).

#### Group 129M-type 2

This group included cases carrying D178N (*n* = 12), E200K (*n* = 4), and 5-OPRI (*n* = 1). As in sCJD [85], this group comprised two histotypes with highly distinctive features, primarily affecting the cerebral cortex or the thalamus. In the former, the spongiform change consisted of large and confluent vacuoles showing a uniform distribution among the neocortices, entorhinal cortex, striatum, and thalamus, with relative sparing of the hippocampus, midbrain, and cerebellum as in sCJD MM2C (Fig. 2e) [85]. Similarly, PrP immunopositivity was typically coarse and distributed around the large vacuoles (perivacuolar pattern) (Fig. 3j, k, and Table 2). As the main difference with sCJD MM2C, all E200K cases uniquely showed “thickened” synaptic PrP deposits in the cerebellar molecular layer, while the 5-OPRI case with the most prolonged disease duration uniquely showed large, patchy, coarse PrP aggregates, often surrounding vacuoles, in the cerebellar molecular layer (Fig. 3l) in addition to the perivacuolar and coarse deposits. These deposits were readily distinguishable from the plaque-like pattern seen in the cerebellar granular layer and white matter of 129V-type 2 cases and from the mini plaque-like aggregates of the 129V-type 1 cases, based on topographic distribution, shape and size. Given the striking analogies with sCJD MM2C, we named this group gCJD 129M-type 2C.

In the second group, which only included participants carrying the D178N-129M haplotype, the lesion profile was highly consistent with that of sCJD MM2T (Fig. 2d). However, given the established correlation between the lesion profile and disease duration in FFI [26], and the longer average disease course in the FFI-129MV group, only the latter showed striking analogies with sCJD MM2T. In contrast, participants of the FFI-129MM group demonstrated a shorter mean disease course (129MM 9.1 ± 1.9 vs. 129MV 26.2 ± 3.9 months, p ≤ 0.0001), a relative sparing of the cerebral neocortex, and less consistent spongiform change (Supplementary Fig. 3, online resource) than those in the FFI-129MV group. As previously reported [26], PrP immunohistochemistry revealed in some of the cases faint synaptic or granular deposits confined to the cerebral cortices and often co-localizing with the spongiform change (Table 2).

#### Group 129M-type “i”

This group included codon 129 heterozygotes carrying the E200K-129M haplotype and showing the PrP^Sc^ type “i” (*n* = 8). Both the morphology and distribution of the spongiform change were somehow reminiscent of gCJD 129V-type 2 and sCJD MV2K (Fig. 2f). The spongiform change featured non-confluent vacuoles of intermediate size and showed a laminar pattern in the cerebral cortex with a predominant involvement of the deeper layers. There was a synaptic pattern of deposition in the cerebellum in all cases. Moreover, as in E200K of the 129V-type 2 group, PrP immunostaining showed intraneuronal, cytoplasmic PrP-immunoreactive globular inclusions in the cerebral cortex's deeper layers, thalamus, cerebellar dentate nucleus, and brainstem nuclei (Fig. 4g).

### Atypical and mixed phenotypes

#### Atypical phenotypes

We applied the term “atypical” when, despite the consistent haplotype and PrP^Sc^ type, the clinicopathological features of gCJD did not match those of the participants (both sporadic and genetic) displaying the same molecular signature. For example, two patients carrying the T183A-129M haplotype associated with PrP^Sc^ type 2 did not match the features of either FFI or gCJD 129M-type 2C. The spongiform change, comprising small, non-confluent vacuoles (Fig. 4h), mainly affected the neocortices, entorhinal cortex, striatum, and thalamus. The hippocampus, midbrain, and, to a lesser extent, the cerebellum were relatively spared by the spongiform change. Immunohistochemistry for PrP revealed granular/mini focal aggregates in the cerebellar molecular layer, striatum, and thalamus (Fig. 4i).

In three patients carrying 5/6-OPRI-129V and PrP^Sc^ types 1 + 2, both the distribution and severity of the neuropathological change were similar to those associated with the T183A mutation (Supplementary Fig. 6, online resource). The spongiform change consisted of non-confluent vacuoles of mixed sizes (small and intermediate) (Fig. 4j). Immunohistochemistry showed PrP aggregates in the form of mini plaque-like, focal deposits in the neocortices. Besides, PrP stripe-like aggregates were detected in the cerebellum molecular layer, along with granular/mini plaque-like deposits (Fig. 4k).

#### Mixed phenotypes

Immunohistochemistry for PrP revealed large, confluent vacuoles and coarse/perivacuolar PrP deposits, hallmarks of PrP^Sc^ type 2 deposition, in 39 out of 127 (30.7%) participants in the 129M-type 1 group. The latter histopathologic features were virtually indistinguishable from those featuring the gCJD 129M-type 2C group and, in most cases, were restricted to focal areas of the occipital cortex. The resulting mixed phenotype was significantly more frequent in participants carrying V210I (48.0%) than in those carrying E200K (23.9%, *p* = 0.025) or other rarer mutations (10.5%, *p* = 0.005), while the comparison with the participants carrying 3–6 OPRI (33.3%) was not statistically significant. The above-mentioned mixed phenotype is virtually indistinguishable from MM(V)1 + 2C, the most frequent subtype combination documented in sCJD [82].

Besides the typical 129M-type 2C features, one participant carrying E200K-129MV showed a moderate to severe spongiform change characterized by non-confluent, intermediate-sized vacuoles in the hippocampus, midbrain, and cerebellum. In these regions, immunohistochemistry revealed plaque-like and pericellular PrP deposits and a strong synaptic positivity, which was more intense in the cerebellar granular layer than in the molecular layer. Altogether, these neuropathological features suggest the co-occurrence of two phenotypes, both associated with PrP^Sc^ type 2 (i.e., 129M-type 2C + 129V-type 2).

Finally, in all the four participants carrying E200K-129M with PrP^Sc^ type 2, we observed “thickened” synaptic PrP deposits in the cerebellar molecular layer. Although in both sporadic and genetic CJD, synaptic PrP in the cerebellar molecular layer is typically associated with PrP^Sc^ type 1, we did not detect PrP^Sc^ type 1 in any of these cases. Therefore, whether these cases represent mixed phenotypes (E200K-129M type 2C + 1) remains to be seen.

### PrP^Sc^ resistance to protease digestion and thermostability

**Fig. 5:**
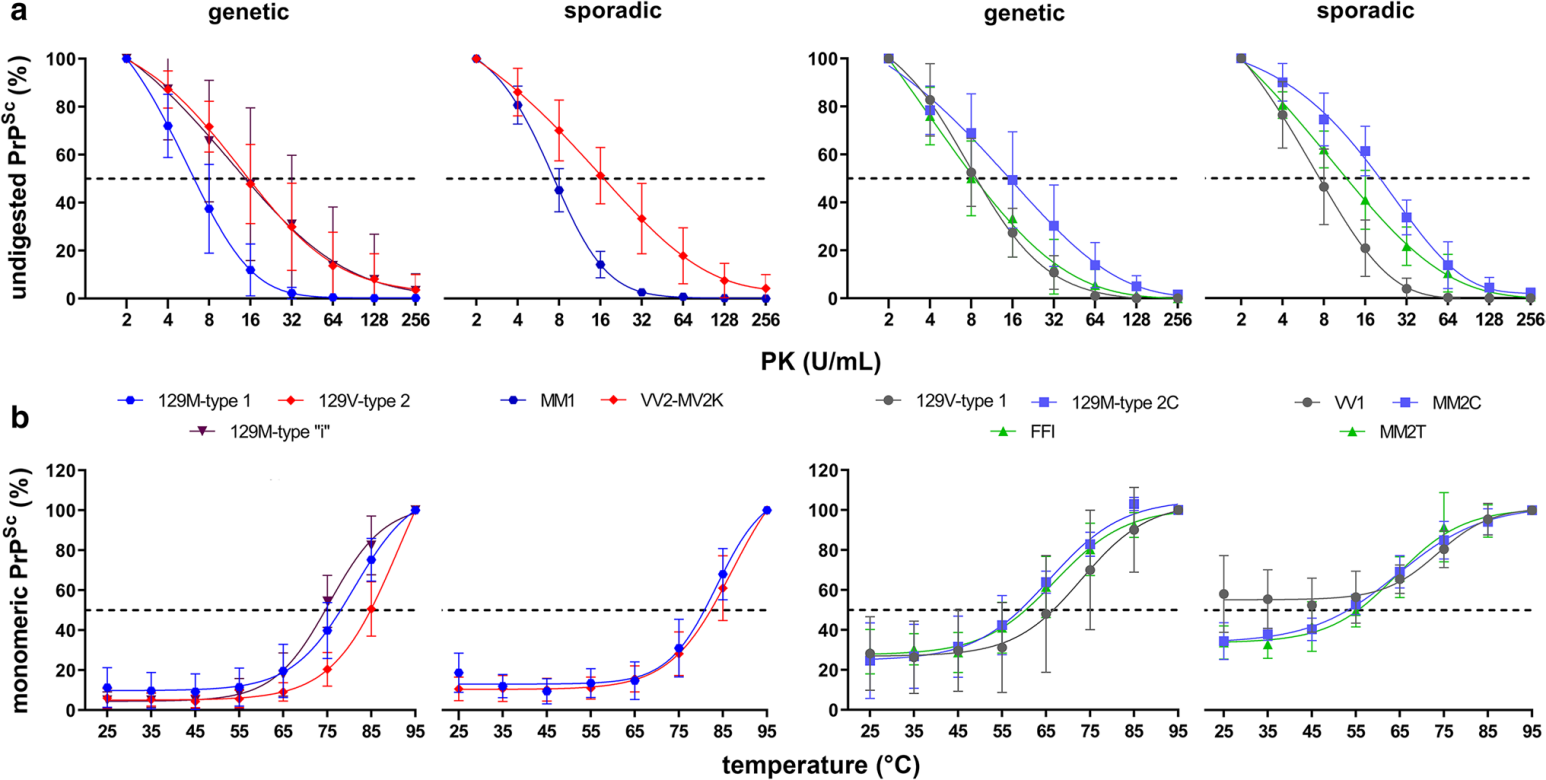
Comparison of PrP^Sc^ biochemical properties between genetic and sporadic CJD and FI. **a, b** PrP^Sc^ PK resistance (**a**) and thermostability (**b**) across the spectrum of genetic and sporadic CJD and FI subtypes. The color and distribution of the curves were adapted to highlight the differences between genetic groups and the similarities with the corresponding sporadic subtypes. The dot lines represent the ED_50_ (PK concentration needed to digest 50% of PrP^Sc^) (**a**) and the T_50_ (temperature needed to solubilize 50% of PrP^Sc^) (**b**)

PrP^Sc^ biochemical properties, such as resistance to PK digestion and thermostability, contributed a molecular signature besides typing to sCJD histotypes [22, 94]. Therefore, we explored whether these PrP^Sc^ features also allow for the reliable differentiation of the gCJD spectrum. PrP^Sc^ resistance to PK digestion was higher in 129V-type 2 cases (ED_50_ 18.0 ± 9.5 U/mL) than in 129M-type 1 (ED_50_ 7.5 ± 2.8 U/mL, p = 0.003) or 129V-type 1 (ED_50_ 8.6 ± 1.1 U/mL p = 0.033) groups, whereas it showed intermediate values in 129M-type 2C and 129M-type “i” cases (ED_50_ 17.2 ± 7.9 U/mL and 15.3 ± 10.8 U/mL, respectively) (Fig. 5a). There were no differences within each group across mutations, except for a slightly lower ED_50_ of PrP^Sc^ type 1 in participants carrying V210I than those carrying E200K (ED_50_ 5.2 ± 0.4 vs. 9.1 ± 2.9 U/mL, *p* = 0.044). Finally, we did not find any significant difference between gCJD and sCJD groups defined by the PrP^Sc^ type/codon 129 genotype combination (Fig. 5a).

After exposure to increasing temperature, PrP^Sc^ in gCJD 129M-type 1 (T_50_ 77.5 ± 4.9 °C), 129V-type 2 (T_50_ 82.9 ± 5.0 °C) and 129M-type “i” (T_50_ 75.2 ± 3.4 °C) cases showed a higher thermostability than in 129V-type 1 (T_50_ 64.8 ± 12.8 °C, vs. 129M-type 1, *p* = 0.002; vs. 129V-type 2, *p* < 0.001) and 129M-type 2C cases (T_50_ 56.9 ± 7.3 °C, vs. 129M-type 1, *p* < 0.001; vs. 129V-type 2, *p* < 0.0001, vs. 129M-type “i”, *p* = 0.006) (Fig. 5b). We did not detect any significant difference between gCJD 129M-type 1, 129V-type 2, 129M-type 2 cases, and the corresponding sCJD subtypes (Fig. 5b). Genetic CJD 129V-type 1 cases showed a higher PrP^Sc^ thermostability than sCJD VV1 (T_50_ 64.8 ± 12.8 vs. < 25.0 °C), which was, at least partially, due to the higher stability of PrP^Sc^ associated with the T188R mutation compared with the D178N variant (T_50_ 77.5 ± 3.4 vs. 57.2 ± 9.3 °C, *p* = 0.036). There were no other significant differences between mutations within each group (Supplementary Fig. 7, online resource).

Finally, PrP^Sc^ associated with FFI cases was highly sensitive to both PK and temperature denaturation. PrP^Sc^ thermostability was similar in FFI and sCJD MM2T groups (T_50_ 60.1 ± 6.5 vs. 57.6 ± 3.2 °C, *p* = 0.394), while PK resistance was slightly lower in the former group (ED_50_ 9.0 ± 0.3.4 vs. 13.2 ± 3.7 U/mL, *p* = 0.093).

### Clinical findings

#### Age at onset and duration of symptoms

The age at onset and disease duration in gCJD also significantly reflected those of their sporadic counterparts. In particular, like sCJD VV1 and MM2T patients, those from the 129V-type 1 and FFI groups were younger than gCJD patients of other groups, while, like sCJD MM1, 129M-type 1 patients had the shortest disease duration (Table 3). A significant exception was observed for participants carrying 5/6-OPRI mutations in the 129M-type 1 group, which were significantly younger at onset and showed a more protracted clinical course than those with other mutations. Similarly, the 5-OPRI case in the 129M-type 2 group showed the most prolonged survival. However, we did not find a similar effect on the age at onset and disease duration in patients of the 129V-type 2 group carrying 5/6-OPRI. The mean age at onset was also slightly less in E200K carriers than in patients carrying all other mutations (61.6 ± 8.9 vs. 65.7 ± 9.7 years, *p* = 0.008) after excluding those with 5/6-OPRI and those of 129V-type 1 and FFI groups. In the 129V-type 1 group, D178N carriers were significantly younger at disease onset than patients with the T188R variant (*p* = 0.001). Furthermore, in the D178N group, 129VV patients were younger at disease onset and showed a trend toward a shorter disease duration than 129VM carriers (Supplementary Table 6, online resource). In the FFI group, 129MV subjects showed a significantly longer disease duration (Supplementary Table 6, online resource). Finally, the two participants carrying the T183A mutation showed a strikingly young age at onset (40 and 41 years) and prolonged disease duration (108 and 48 months).

**Table 3:** Demographic findings in the gCJD and FFI groups and comparison with sCJD subtypes

			Age at onset (years)			Disease duration (months)		
	*n*	*F*, %	Genetic	Sporadic	*p*	Genetic	Sporadic	*p*
**129M-type 1**				**MM(V)1**			**MM(V)1**	
Overall	141	44.7	64.3±10.4^a,b^	69.2±9.2	< 0.0001	7.5 ± 16.8^c-e^	4.1±3.8	0.0318
E200K	58	44.8	62.1±9.4^g,k,l^	“	<0.0001	4.5±2.7^h,m,n^	“	ns
V210I	51	37.3	64.6±9.7^h^	“	0.0392	3.8±3.3^h^	“	ns
3/4-OPRI	6	33.3	65.0±3.9^g^	“	ns	8.6±4.6^h^	“	ns
5/6-OPRI	6	50.0	45.8±7.9^i,o^	“	<0.0001	65.7±54.3^i,p^	“	<0.0001
Others A^§^	20	65.0	68.3±11.3	“	ns	7.4±8.2	“	ns
**129V-type 2**				**VV2/MV2K**			**VV2/MV2K**	
Overall	17	76.5	63.5±8.2^f^	64.5±11.9	ns	9.0±6.6	9.8±8.3	ns
E200K	5	80.0	58.8±8.0	“	ns	7.0±3.8	"	ns
5/6-OPRI	9	88.9	65.0±8.9	“	ns	8.3±4.8	"	ns
Others B^¶^	3	33.3	66.7±4.0	“	ns	19.3±11.7	"	ns
**129V-type 1**				**VV1**			**VV1**	
Overall	19	47.4	51.2±11.3	42.0±10.2	ns	17.4±9.6	18.4±3.4	ns
D178N	13	53.8	45.7±6.2^j,q^	“	ns	20.2±10.1	“	ns
T188R	6	33.3	64.4±9.9	“	0.0009	10.8±2.7	“	ns
**129M-type 2T (FFI)**				**MM2T**			**MM2T**	
D178N	13	46.1	54.4±8.9	42.0±10.3	0.0155	16.2±9.2	18.2±5.1	ns
**129M-type 2C**				**MM2C**			**MM2C**	
Overall	5	60.0	62.4±7.1	61.5±16.4	ns	30.4±37.6	17.9±8.5	ns
E200K	4	50.0	61.7±8.1	“	ns	14.0±9.4	“	ns
5-OPRI	1	100	65	“	–	96	“	–
**129M-type “i”**								
E200K	8	50.0	60.1±7.0	**–**	**–**	16.5±15.1	**–**	**–**
Atypical								
T183A-129M	2	0.0	40.5±0.7	**–**	**–**	78.0±42.4	**–**	**–**
5/6-OPRI-129V	3	100	58.7±10.4	**–**	**–**	11.7±0.6	**–**	**–**

The names of histo-molecular groups in both genetic and sporadic CJD are in bold

^§^Others A includes: R208H, *n* = 7; V203I, *n* = 3; E196A/K, *n* = 6; T188K, *n* = 1; R148H, *n* = 1; D211Q, *n* = 2

^¶^Others B includes R208H, *n* = 1; E196K, *n* = 1; T188A, *n* = 1. The 3/4-OPRI group includes both 3-OPRI and 4-OPRI, 5/6-OPRI includes both 5-OPRI and 6-OPRI cases

^a^^−^^f^Statistically significant comparisons between genetic groups (overall data): ^a^vs. 129V-type 1, *p* ≤ 0.001. ^b^vs. FFI, *p* ≤ 0.05. ^c^vs. 129V-type 1, *p* ≤ 0.0001. ^d^vs. FFI, *p* ≤ 0.0001. ^e^vs. 129M-type “i” *p* ≤ 0.01. ^f^vs. 129V-type 1, *p* ≤ 0.05

^g−j^Statistically significant comparisons between *PRPN* mutations within each genetic group: ^*g*^vs. 129M-type 1 5/6-OPRI, *p* ≤ 0.01. ^h^Compared to 129M-type 1 5/6-OPRI, *p* ≤ 0.0001. ^i^Compared to 129M-type 1 Others A, p ≤ 0.0001. ^j^Compared to 129V-type 1 T188R, *p* ≤ 0.001

^k−q^Statistically significant comparisons between carriers of the same *PRNP* mutation belonging to different groups: ^k^vs. 129M-type 2C E200K, *p* ≤ 0.001. ^l^vs. 129M-type “i” E200K, *p* ≤ 0.001. ^m^vs. 129M-type 2C E200K, *p* ≤ 0.01. ^n^vs. 129M-type “i” E200K, *p* ≤ 0.01. ^o^vs. 129V-type 2 5/6-OPRI, *p* ≤ 0.01. ^p^vs. 129V-type 2 5/6-OPRI, *p* ≤ 0.05, ^q^vs. FFI, *p* ≤ 0.01

#### Clinical symptoms and signs at onset

Genetic CJD 129V-type 2 and 129M-type “i” groups presented with ataxia in most cases (70%). In contrast, 129V-type 1 and 129M-type 2C groups showed cognitive impairment as the most frequent presenting sign (82% and 75% of cases, respectively) (Table 4).

**Table 4:** Main symptoms and signs at disease onset in gCJD groups and comparison with sCJD subtypes

Phenotype	g/s	n	Cognitive^#^ (%)	*p*	Ataxia/ cerebellar (%)	*p*	Visual^§^ (%)	*p*	Myoclonus (%)	*p*	Unilateral (%)	*p*
129M-type 1	g	138	79 (57.2)	0.0042	69^a^^,b^ (50.0)	ns	25 (18.1)	0.0241	3 (2.2)	ns	23 (16.7)	ns
MM(V)1	s	127	94 (74.0)		52 (40.9)		38 (29.9)		5 (3.9)		18 (14.2)	
129V-type 2	g	14	6 (42.9)	ns	11^c^ (78.6)	ns	0	–	0	–	0	–
VV2	s	45	14 (31.1)		43 (95.5)		0		0		1 (2.2)	
MV2K	s	26	14 (53.8)		22 (84.6)		1 (3.8)		0		1 (3.8)	
129V-type 1	g	11	9 (81.8)	ns	1 (9.1)	–	0	–	0	–	0	–
VV1	s	5	5 (100)		0		0		0		0	
129M-type 2C	g	4	3 (75.0)	ns	0	–	0	–	0	–	0	–
MM2C	s	13	12 (92.3)		0		2 (15.4)		0		1 (7.7)	

*g/s* genetic/sporadic CJD, *ns* not significant

^#^One or more of: memory loss, aphasia, confusion and/or disorientation, intellectual decline

^§^One or more of: visual loss, visual field defect, visual distortion, abnormal color vision, cortical blindness

^a^^−^^c^Statistically significant comparisons between genetic groups: ^a^vs. 129V-type 2 ≤ 0.05, ^b^vs. 129V-type 1 ≤ 0.05; ^c^vs. 129V-type 1 ≤ 0.001

Both cognitive decline and ataxia characterized the clinical onset in approximately half of the patients with 129M-type 1, without significant differences among *PRNP* mutations (Supplementary Table 7, online resource). Moreover, in about 20% of 129M-type 1 cases, the disease presented with unilateral neurological signs and visual symptoms. We also found a higher prevalence of isolated memory loss in the gCJD groups characterized by remarkable hippocampal spongiform change and gliosis (129V-type 2, 129V-type 1, and 129M-type “i”) (data not shown).

#### Clinical symptoms and signs during evolution

A variable combination of cognitive, behavioral/psychiatric, cerebellar, pyramidal, and extrapyramidal signs characterized the clinical course of most gCJD cases (Supplementary Table 8, online resource). However, similar to sCJD, visual symptoms were more frequent in the 129M-type 1 group than in other groups, whereas all 129V-type 2 patients presented with cerebellar symptoms or signs. Myoclonus was frequent in gCJD groups with predominant cortical involvement (129M-type 1, 129V-type 1, and 129M-type 2C), while it occurred less frequently and, possibly, at a later stage in the other groups.

#### Diagnostic investigations

CSF prion RT-QuIC demonstrated positive PrP^Sc^ seeding activity in 92.1% of the tested gCJD patients (35/38) and had a higher sensitivity than CSF t-tau (41/46, 89.1%) and 14-3-3 proteins (95/113, 84.1%) (Supplementary Table 9, online resource). Fifty-five out of 67 cases (82.1%) presented with definite signal hyperintensities in FLAIR and/or DWI-MRI sequences. Increased signal more frequently involved the striatum than neocortices (83.6% vs. 41.8% of cases) (Supplementary Table 10, online resource). Finally, EEG recordings disclosed diffuse, periodic sharp-wave complexes, a feature supporting the diagnosis of CJD, in 89 out of 167 cases (53.3%). The latter finding was most frequent in 129M-type 1 than in other gCJD groups (Supplementary Table 11, online resource). Further details about the results of diagnostic investigations are provided in the Supplementary Materials, online resource.

## Discussion

To reach a comprehensive characterization of the gCJD phenotypic spectrum, we carried out for the first time a systematic analysis of biochemical, molecular, clinical, and histopathological features in a large cohort, including 17 different *PRNP* mutations that represented the most frequent pathogenic human *PRNP* mutations linked to gCJD. The added value of the present analysis compared to previous studies include:

- The deepest characterization of the disease subtypes linked to the most prevalent human *PRNP* mutation (E200K), including the description of two previously unreported histo-molecular phenotypes (i.e., M2C-E200K and M”i”-E200K).
- The definition of the phenotypic spectrum of gCJD V210I in the largest patient population gathered to date.
- The phenotypic characterization of 27 previously unreported individuals carrying five different mutations in association with PrP^Sc^ type 2, which is extremely rare in gCJD, especially in combination with the codon 129M genotype.
- The systematic analysis of the co-occurrence and regional distribution of PrP^Sc^ types 1 and 2 in multiple brain regions in 142 individuals with gCJD.
- The characterization of PrP^Sc^ resistance to protease digestion and temperature denaturation in gCJD.
- A systematic comparison of data obtained with the same methodology in two large cohorts of gCJD and sCJD.

Overall, the results provide an accurate definition and classification of gCJD subtypes and insights into significant aspects of human prion biology, such as the effect of *PRNP* variants on phenotypic expression and the origin of prion strains.

Our findings demonstrate a striking parallelism between genetic and sporadic CJD phenotypes [20]. While overall the combination of *PRNP* genotype and PrP^Sc^ type accounts for the ultimate clinicopathological phenotype in both the forms, the genotypic variability determined by most *PRNP* mutations has only a moderate or even minimal effect on the disease phenotype. Our finding of divergent phenotypes in participants carrying the same *PRNP* mutation in *cis* with distinct codon 129 genotypes in patients carrying the E200K, 5/6-OPRI, D178N, R208H, and E196K variants underlines the central role of codon 129 (on the mutated allele). Moreover, the detection of distinct PrP^Sc^ types (1, 2 or “i”) in participants carrying the same *PRNP* haplotype (E200K-129M, 5-OPRI-129M) firmly demonstrates that PrP^Sc^ can assume distinct conformations in individuals carrying the same *PRNP* sequence, even in the presence of a mutation. Finally, the fact that in gCJD, most mutations are preferentially associated with PrP^Sc^ type 1 when in *cis* with 129M, and with type 2 when in *cis* with 129V, further proves the similarities with sCJD. These findings strengthen the notion that in gCJD, as in sporadic and acquired CJD forms, the codon 129 genotype plays a fundamental role in directing PrP^Sc^ abnormal conformation and the emergence of a specific prion strain [30, 34, 44, 50, 63, 68, 93].

Our data indicate that specific mutations have a variable effect on phenotypic expression. Specifically, four groups can be distinguished. The first would include several *PRNP* variants linked to PrP^Sc^ type 1 and 129M in the mutated allele with apparently incomplete penetrance and no effect on PrP^Sc^ glycotype (e.g., V210I and V203I). These mutations have virtually no effect on the clinicopathological disease phenotype. The second group would include some *PRNP* variants know to have a high penetrance (E200K and OPRI) with or without a specific effect on PrP^Sc^ glycotype. Examples of the effect of these mutations on the pathological phenotype include the “thickened” synaptic pattern of PrP deposition, which is best seen in the molecular layer of cerebellum, the stripe-like granular deposits perpendicular to the surface of the cerebellar molecular layer, which are hallmarks of the E200K and 5/6-OPRI, respectively, and the intraneuronal globular deposits found in the V2-E200K and M”i”-E200K groups [14, 16, 41, 53, 61, 62]. Similar effects are the young age at onset and the relatively long disease duration in 5/6-OPRI cases, at least when combined with 129M and PrP^Sc^ type 1. The third group would include mutations like T183A located near the PrP glycosylation sites, which, by blocking the addition of the first glycan chain to residue 181 [17], profoundly affect the PrP^Sc^ glycoform profile and the resulting disease phenotype. Indeed, the T183A-129M haplotype has been linked to PrP^Sc^ type 2 with an atypical glycoform ratio and a histotype lacking several pathological distinctive features of the sCJD MM2 phenotypes, with whom it shares the 129M-type 2 combination. The observation is consistent with the results of experimental transmission studies demonstrating that the glycosylation status of host PrP^C^ affects prion strain characteristics [15]. Finally, although D178N and T188R mutations do not induce a novel phenotype, they share the peculiarity of an association with molecular types, which are extremely rare in sCJD. Notably, only the rare 129M-type 2 or 129V-type1 combinations have been detected to date in patients carrying the D178N mutation, making these combinations more common in genetic prion diseases than in the sporadic form.

In this study, we focused on *PRNP* mutations linked to CJD or FFI, representing only a subset of the inherited prion diseases, albeit the most common. Consequently, it would be misleading to extend the conclusions driven by the present findings to the inherited PrP-amyloidosis. Indeed, the absence of a definite sporadic counterpart strongly suggests that, in contrast to CJD and FFI, phenotypic diversity in GSS and related syndromes is primarily driven by the *PRNP* mutation. However, the recent characterization of variably protease-sensitive prionopathy, a novel phenotype of sporadic prion disease associated with distinctive PrP^Sc^ properties and clinico-pathological features, has clearly shown that the formation of C-terminally truncated, unglycosylated and anchorless fragments, thought to be GSS-specific until recently, may also occur in sporadic prion disease in the absence of any mutation [102], a finding which supports the view that most PrP^Sc^ conformational diversity may develop independent of the presence of *PRNP* mutations.

To provide an updated definition of the spectrum of gCJD variants accounting for all the major molecular/genetic determinants of the phenotype, we suggest a novel terminology that, in a hierarchical order, include: a) the *PRNP* genotype at codon 129 in *cis* with the mutation, b) the type of PrP^Sc^, and c) the *PRNP* mutation to propose a classification of gCJD in six main variants or subtypes (see Table 5), that largely, but not entirely, reproduces those already defined in sCJD. Additionally, we propose to provisionally classify the few cases not fulfilling the six groups as “atypical”.

**Table 5:** Nomenclature and classification of gCJD subtypes

gCJD subtype	Mean duration (months)	Neuropathological features	Major differences with sCJD subtypes
M1-mutation	4.6^a^	Spongiform change (microvacuoles) mainly affecting the cerebral cortex, striatum, thalamus and cerebellum; often prominent involvement of occipital cortex; “synaptic type” PrP staining with mutation-specific effects in OPRI and E200K carriers	Analogous sCJD subtype: MM(V)1 Younger age at onset and longer disease duration in 5/6-OPRI. Mutation-specific PrP deposits: “thickened” synaptic (E200K, E196A/K), cerebellar stripes (OPRI) in molecular layer
V2 (K)-mutation with or without kuru plaques	9.9	Prominent involvement of diencephalon, basal ganglia and cerebellum; in neocortex, spongiform change (intermediate vacuoles) often limited to deep layers (laminar distribution); PrP “plaque-like”, focal deposits mainly in the cerebellar granular layer and thalamus; amyloid-kuru plaques in the cerebellum in a subgroup of cases	Analogous sCJD subtype: VV2 and MV2K Longer disease duration in 129VV carriers. Absence of cortical plaque-like deposits in E200K and of cerebellar kuru plaques in E200K-129VM. Mutation-specific PrP deposits: “thickened” synaptic and globular intraneuronal (E200K), cerebellar stripes (OPRI), cerebellar kuru plaques in R208H-129VV
V1-mutation	17.4	Spongiform change (intermediate vacuoles) in the cerebral cortex, hippocampus and striatum; brainstem and cerebellum spared; “mini plaque-like” PrP deposits in the cerebellar molecular layer and deep cortical layers	Analogous sCJD subtype: VV1 More consistent “mini plaque-like” PrP deposits
M2C-mutation *(cortical variant)*	14.0	Spongiform change (large confluent vacuoles) with “perivacuolar/coarse” PrP deposits in the neocortices; cerebellum relatively spared	Analogous sCJD subtype: MM(V)2C No major differences. Mutation-specific PrP deposits: "thickened" synaptic (E200K)
FFI or M2T-D178N *(thalamic variant)*	16.2	Prominent atrophy of the medial thalamus and inferior olivary nuclei, spongiform change may be absent (129MM) or patchy (129MV), and usually only involves the cerebral cortex	Analogous sCJD subtype: MM2T Disease duration and spongiform change influenced by codon 129 genotype of wild-type allele in FFI. Phenotype of the sporadic form (all 129MM) resembling FFI-129MV
M”ì”-E200K	16.5	Distribution of neuropathological change similar to the V2-mutation group; globular intraneuronal PrP deposits	No analogous sCJD subtype

^a^M1-5/6-OPRI were excluded from the analysis of disease duration due to their mutation-specific effect

The classification recognizes the prion strains as the critical molecular determinants of the phenotypic spectrum of all forms of CJD. Transmission studies conducted to date with sCJD inocula currently support the existence of five distinct strains of CJD prions: the M1 related to the typical CJD phenotype or myoclonic variant [MM(V)1], V2 linked to the ataxic and kuru-plaque variants (VV2 and MV2K), M2T causing the thalamic variant (MM2T or sporadic FI), M2C linked to the cortical MM2C subtype, and V1 associated with the VV1 subtype [8, 13, 42, 74, 79]. Transmission data for gCJD and FFI are available for the E200K-129M [4, 51, 71, 84] and V210I-129M [59] haplotypes (expressing PrP^Sc^ type 1), a few cases carrying insertion mutations coupled with M at codon 129 in the mutated allele [61], a single case carrying the E200K-129V haplotype [4], and several FFI cases [25, 58, 97, 98]. Therefore, the limited data gathered to date demonstrating the M1 and M2T strains in gCJD and FFI are in line with the idea that the spectrum of strain variation in gCJD significantly parallels that identified in sCJD. The data we contributed with the present study further corroborates this notion by strongly supporting the occurrence of V2, V1, and M2C strains in gCJD, in addition to M1 and M2T. However, further transmission studies will be needed to fully confirm our conclusions on the parallelism between gCJD and sCJD regarding the spectrum of prion strains.

Of note, the V2 strain is the one showing the most significant differences between the genetic and sporadic forms. Transmission studies have to date linked the V2 strain to CJD subtypes VV2 and MV2K, occurring in both sporadic and acquired forms of the prion disease [46, 48, 84, 88], although some authors recently argued that the MV2K subtype should be considered a distinct prion “strain” based on the unique PrP^Sc^ and histopathologic features in the natural host [70]. Remarkably, an additional subtype, named MM “i”K, which only affected patients with iatrogenic CJD showed the same transmission properties of the VV2 and MV2K subtypes in humanized knock-in transgenic mice [46, 48]. The existence of the latter subtype makes V2 the only strain affecting all the three codon 129 genotypes, although it preferentially converts and replicates the PrP^C^-129V [8, 46, 70]. As an additional peculiarity, the PrP^Sc^ conformation associated with the V2 strain changes according to the presence of V or M at position 129 [49, 84], resulting in a PK-resistant core of 19 kDa (type 2) in the patients carrying VV, a 20 kDa fragment (type “i”) in those with MM, and a “doublet” including both PrP^Sc^ fragments in those carrying MV [45, 72]. However, only patients with 5/6-OPRI-129V(M) showed a genuine MV2K histotype, including a western blot profile with the 20 and 21 kDa doublet. In contrast, those carrying E200K-129V(M), despite the similar lesion profile lacked the kuru-type amyloid plaques. The relative contribution of the wild-type and mutated alleles to PrP^Sc^ formation might explain the two mutations' different behavior: in patients carrying the E200K mutation, only the mutated allele contributes to PrP^Sc^ formation, whereas both mutant and wild-type PrP^C^ convert to PrP^Sc^ in OPRI [23, 28].

The identification of the novel M“i”-E200K gCJD phenotype requires further comments. Both the clinicopathological and molecular data strongly suggest that also this histotype is linked to the V2 strain. Firstly, the lesion profile and other histopathological features of this histotype strikingly resemble those of the V2-E200K group. Moreover, both PK resistance and thermo-solubility of M“i”-E200K PrP^Sc^ show more similarities to those of the V2-E200K PrP^Sc^ than to those of M1-E200K PrP^Sc^. These findings, combined with the notion that PrP^Sc^ type “i” (129M) has been linked to V2 prions in iatrogenic CJD, strongly support the idea that the M“i”-E200K phenotype reflects the adaptation of V2 strain to the 129M-E200K host genotype.

To explain why the 129M/type “i” combination does not have a sporadic counterpart, we hypothesized that the E200K mutation might act as a permissive factor for the V2 adaptation through a change in conformation or by favoring the selection of highly glycosylated isoforms [6]. The lack of kuru plaques in M”i”-E200K might, again, depend on the mono-allelic origin of PrP^Sc^ (from the mutant PrP allele), somehow preventing the interaction between PrP isoforms generated by different alleles, or, again on a mutation-specific effect, possibly related to the glycan chains inhibiting PrP^Sc^-amyloid plaque formation. Experimental transmission studies are required to explore these issues further. Finally, given that all M“i”-E200K affected participants carried 129V in *trans* with mutation, we cannot rule out a possible role of the wild-type allele on the emergence of this phenotype.

Another interesting finding was the presence of intraneuronal cytoplasmic PrP inclusions in a subgroup of patients carrying the E200K mutation. Intraneuronal PrP immunoreactivity has been consistently observed in animals but not in human prion diseases [43]. Interestingly, Kovacs et al. [54] reported that a particular type of intraneuronal PrP immunoreactivity, they named type III, resembling the "globular" pattern we observed, distinguishes a subset of gCJD E200K individuals. In contrast, the presence of smaller dot-like intraneuronal PrP deposits characterized both genetic and sporadic CJD brains. Although, we could not replicate the whole spectrum of intraneuronal deposits described by Kovacs et al. [54], likely because of differences in the PrP immunostaining protocol, our findings are consistent with the intraneuronal globular inclusions being a specific feature of the molecular subtypes V2-E200K and M"i"-E200K linked to the V2 strain. The reason why the E200K mutation favors the formation of intracellular PrP deposits that are nor readily seen in sCJD might depend on the abnormal intracellular processing of the mutant PrP showing impaired transport to the cell surface [18].

By examining this large cohort of gCJD cases, we also detected rare “atypical” phenotypes, difficult to reconcile with one of the major histotypes included in the current sCJD classification. Notably, atypical phenotypes shared a PrP^Sc^ isoform characterized by a marked under-representation of the diglycosylated isoform. Furthermore, they showed an exclusive or predominant PrP^Sc^ type 2 migration profile. It is worth noting that a similar immunoblot profile also characterizes some sCJD with atypical features that remain unclassified to date. The lesion profiles and the PrP patterns of deposition were comparable between haplotypes suggesting that specific PrP^Sc^ properties, such as the glycotype, contributed to the phenotype more than the haplotype. The transmission of these cases would determine whether the atypical phenotype is driven by a novel prion strain or by the host genotype.

Another interesting phenomenon determining phenotypic diversity in prion disease is the coexistence of more than one prion strain in the natural host. We have previously shown [82] that the mixed phenotypes resulting from the co-occurrence of PrP^Sc^ types 1 and 2 affect about 35% of sporadic CJD cases, although other studies reported a wide range of estimates of the phenomenon likely due to differences in detection methodologies [36, 47, 86]. All 3 codon 129 genotypes may be affected although MM cases are more frequently involved, given that coexistence of the MM(V)1 and MM2C subtypes is the most prevalent association, involving almost 40% of MM cases [82]. The present results provide evidence that the mixed phenotypes also characterize the gCJD spectrum. They essentially reproduce the most common mixed variants previously described in sCJD [82]. However, when compared using the same protocol and methodology, the overall frequency appears slightly lower than in sCJD (21% vs. 35%), an effect that is, once again, mutation dependent. Specifically, mixed phenotypes were extremely rare in the M1-E200K group despite the large number of patients analyzed. In contrast, patients with M1-V210I showed a slightly higher prevalence compared to the corresponding sCJD group [82]. Altogether, these findings suggest that specific *PRNP* mutations, particularly those significantly affecting the disease phenotype like the E200K, may modulate disease susceptibility in a strain-specific manner. Accordingly, the E200K mutation would determine a reduced relative M2 vs M1 susceptibility. In contrast, *PRNP* mutations with incomplete penetrance such as the V210I, would be more permissive to reproduce the same prevalence and type of multiple stains/phenotype coexistence we observed in the sporadic form.

The age at onset in gCJD is another issue requiring specific comments. On an average, it is generally believed that the disease starts earlier in patients with gCJD compared to sCJD owing to the specific predisposing effect of the mutations [67]. However, our data depict a more complex picture, which underlines the role of the prion strain in both sCJD and gCJD, irrespective of the presence of a *PRNP* mutation. We have previously shown that the disease onset in sCJD occurs significantly earlier in sCJD VV1 and MM2T than in the other subtypes [1, 82]. Notably, we observed the same difference in the age at onset between the 129V-type 1, and FFI groups and most of the other gCJD groups. Exceptions concerned the *PRNP* mutations influencing PrP^Sc^ properties and disease phenotype, including E200K, T183A, and 5/6-OPRI, albeit to a different extent. Compared to sCJD, T183A and 5/6-OPRI-129M carriers showed a remarkably younger age at onset, whereas it was only slightly earlier, independent of the haplotype/PrP^Sc^ type combination, in those carrying E200K. Of note, 5/6-OPRI in combination with 129V cases did not show the same effect on age at onset, suggesting a strain-specific (earlier onset in M1 but not V2 affected participants) effect.

Strengths of our study include the comprehensive analysis of the molecular, histopathological, and, to a lesser extent, clinical data of the largest cohort of gCJD cases examined to date, and the direct comparison with a series of sCJD cases representative of currently identified disease subtypes. Limitations of this study include the absence of few rare mutations (e.g., G114V, E200G, and V189I) that have been linked to a CJD phenotype, and the small/imbalanced sample sizes of patient subgroups that are virtually inevitable, given the highly heterogeneous prevalence of the *PRNP* variants and phenotypes, which is also limiting the power of the statistical comparison within each of the gCJD groups, especially for demographic data and clinical features.

In conclusion, the results of the present study confirm and significantly extend previous data on gCJD collected in small case series or case reports. The results support an updated classification of gCJD in six groups or subtypes that largely, but not entirely, reproduce those already defined in sCJD. The most significant divergence between the two forms includes the 129M-type “i” combination in patients carrying the E200K variant, likely representing the adaptation of the V2 CJD strain to the 129M-E200K allele, which has no counterpart in the sporadic form. Additionally, some mutations significantly affect the morphology and cellular distribution of PrP deposits (E200K, OPRI, E211Q-129M, and R208H-129V) or, rarely, are linked to atypical phenotypes (T183A-129M). The latter features introduce further complexity to the histopathologic classification of CJD subtypes but may also help neuropathologists to suspect the genetic etiology of disease through the identification of mutation-specific patterns of PrP deposition.

Overall, our data show that most *PRNP* mutations have limited or even no effect on phenotypic expression compared to the PrP^Sc^ type/codon 129 (mutated allele) combination; however, they may act in conjunction with codon 129 genotype in modulating disease susceptibility to a specific PrP^Sc^ type/prion strain. They apparently do not generate novel, mutation-specific conformations, a conclusion that must be confirmed through experimental transmission studies.

## Supplementary Information

Below is the link to the electronic supplementary material.

Supplementary file1 (PDF 1287 kb)
