# Supplementary material for: Phenotypic diversity of genetic Creutzfeldt–Jakob disease: a histo-molecular-based classification
Source: Acta Neuropathol. 2021 Jul 29;142(4):707–28. doi: 10.1007/s00401-021-02350-y (PMC8423680; doi:10.1007/s00401-021-02350-y)
Supplement: Supplementary file 1 — Supplementary file1 (PDF 1287 kb) [file 401_2021_2350_MOESM1_ESM.pdf]

Supplementary Data

Phenotypic diversity of genetic Creutzfeldt-Jakob disease: A histo-molecular-based classification

Simone Baiardi, Marcello Rossi, Angela Mammana, Brian S. Appleby, Marcelo A. Barria, Ignazio Calì, Pierluigi Gambetti, Ellen Gelpi, Armin Giese, Bernardino Ghetti, Jochen Herms, Anna Ladogana, Jacqueline Mikol, Suvankar Pal, Diane L. Ritchie, Viktoria Ruf, Otto Windl, Sabina Capellari, Piero Parchi.

Corresponding authors: Prof. Piero Parchi and Prof. Sabina Capellari, IRCCS Istituto delle Scienze Neurologiche, Ospedale Bellaria, Via Altura 1/8, 40139 Bologna, Italy. E-mails: piero.parchi@unibo.it, sabina.capellari@unibo.it. Phone: +39-051-4966115. Fax: +39-051-4966208.

Journal: Acta Neuropathologica

Table of Contents

**Supplementary methods**	**3**
Addendum to patient selection	3
Diagnostic investigations	3
Data analysis	3
**Supplementary results**	**4**
CSF analyses	4
Brain magnetic resonance imaging (MRI)	4
Electroencephalogram	4
**Supplementary Table 1**	**6**
**Supplementary Table 2**	**7**
**Supplementary Table 3**	**8**
**Supplementary Table 4**	**9**
**Supplementary Table 5**	**10**
**Supplementary Table 6**	**11**
**Supplementary Table 7**	**12**
**Supplementary Table 8**	**13**
**Supplementary Table 9**	**14**
**Supplementary Table 10**	**15**
**Supplementary Table 11**	**16**
**Supplementary Fig. 1**	**17**
**Supplementary Fig. 2**	**18**
**Supplementary Fig. 3**	**19**
**Supplementary Fig. 4**	**20**
**Supplementary Fig. 5**	**21**
**Supplementary Fig. 6**	**22**
**Supplementary Fig. 7**	**23**
**Supplementary references**	**24**

SUPPLEMENTARY METHODS

Addendum to patient selection

One hundred and fifty-one patients died in Europe and 57 in the USA. European cases were from the Laboratory of Neuropathology at the Institute of Neurological Science of Bologna (ISNB), Italy (n=98), the Center for Neuropathology and Prion Research of the Ludwig-Maximilians-Universität, Munich, Germany (n=36), the National CJD Research & Surveillance Unit (NCJDRSU), University of Edinburgh, Edinburgh, United Kingdom (n=10), the Lariboisière Hospital, Paris University, Paris, France (n=6), and the Medical University of Vienna, Vienna, Austria (n=1). Thirty-nine cases were from the National Prion Disease Pathology Surveillance Center (NPDPSC) of the United States, Cleveland, USA, and 7 from the Department of Pathology and Laboratory Medicine, Indiana University School of Medicine, Indianapolis, USA. Finally, 11 subjects belonged to the National Institutes of Health series of transmitted cases [1].

Diagnostic investigations

We classified electroencephalographic (EEG) recording abnormalities into the following categories: i. diffuse non-specific slowing, ii. paroxysmal discharges (PDs), and iii. periodic sharp-waves complexes (PSWCs) [8]. We considered only magnetic resonance imaging (MRI) studies, including fluid-attenuated inversion recovery (FLAIR) and/or diffusion-weighted imaging (DWI) sequences. According to current diagnostic criteria for sCJD [4], we considered a “typical” finding the presence of hyperintensities on T2-FLAIR and/or DWI in at least two cortical regions (temporal, parietal, and occipital cortices) and/or the striatum. Standard SDS PAGE and immunoblotting were used for detection of protein 14-3-3 in the cerebrospinal fluid (CSF), whereas total-tau (t-tau) protein levels were measured by quantitative ELISA (pathological cut-off value >1250 pg/mL) [5]. At ISNB, NPDPSC, and NCJDRSU, the CSF prion real-time quaking induced conversion (RT-QuIC) assay was performed using either full-length (Ha23-231) or truncated (Ha90-231) hamster recombinant PrP^{C} (or both) as substrate according to previously published protocols [2, 3, 5, 6].

Data analysis

Statistical analysis was performed using GraphPad Prism 8 (GraphPad Software, La Jolla, CA, USA). Depending on the data distribution, the Mann–Whitney *U*-test or the two-tailed Student *t*-test were used, as appropriate, to test differences between two groups of continuous variables. In contrast, one-way ANOVA or Kruskall-Wallis test was used for multiple group comparisons. The chi-square test and Fisher’s exact test were adopted for categorical variables. A *p-value* of <0.05 was considered statistically significant.

SUPPLEMENTARY RESULTS

CSF analyses

Overall, CSF analyses showed a higher sensitivity than MRI and EEG in the identification of gCJD patients. CSF was collected by a lumbar puncture on average at 3.1±2.6 months from onset (range 0.5-12 months). Western blot assay for 14-3-3 protein was performed in 113 cases and gave positive results in 95 (84.1% sensitivity). The assay's sensitivity was greater than 65% for all gCJD-associated mutations, but the T188R variant (129V-type 1 group) yielding negative results in 2 out of 3 tested participants (33.3% sensitivity, Supplementary Table 9). T-tau ELISA revealed values above threshold (i.e. >1250 pg/ml) in forty-one out of 46 cases (89.1%). Patients with t-tau <1250 pg/ml included 1 V2-E200K, 2 M"i"-E200K, and 2 subjects with OPRIs (Supplementary Table 9). CSF prion RT-QuIC demonstrated positive PrP^{Sc} seeding activity in 35 out 38 cases (92.1% sensitivity). Compared with CSF surrogate biomarker of neurodegeneration (t-tau and 14-3-3 proteins), RT-QuIC showed a higher sensitivity independently from the protocol used (i.e., first-generation PQ-protocol vs. second-generation IQ-protocol). Interestingly, negative results in the prevalent groups were invariably associated with the PQ protocol. Moreover, like in sCJD VV1, the RT-QuIC showed a low sensitivity (33.3%) in the gCJD 129V-type 1 group.

Brain magnetic resonance imaging (MRI)

Results from 67 brain MRI including at least one sequence between FLAIR and DWI have been considered in the analysis. Patients underwent cerebral MRI on average 3.3±4.0 months from clinical onset (range 0.5-23 months). Fifty-five out of 67 cases (82.1%) presented with "typical" signal hyperintensities involving the striatum and/or neocortices, the former being more frequently affected (85.5% vs. 45.4%, p<0.0001). Despite the small sample size, a suboptimal sensitivity has been detected in 129V-type 2 and 129M-type "i" groups (55.6% and 50.0%, respectively, Supplementary Table 10). In the latter groups, hyperintensities involved only the striatum, whereas neocortices were invariably spared.

Electroencephalogram

EEG recordings were performed on average at 3.2±3.4 months from the clinical onset (range 0.5-23 months). Like in sCJD, the EEG findings in gCJD had the lowest sensitivity among the investigations included in the diagnostic criteria for CJD. Indeed, the detection of periodic sharp-wave complexes (PSWCs), a feature supporting CJD diagnosis, was limited to 53.3% of gCJD cases. The remaining half of patients showed unspecific, diffuse slowing or, to a lesser extent, paroxysmal EEG activity. Paralleling sCJD MM(V)1, PSWCs

were a relatively common finding in gCJD 129M-type 1 (62.9%) as compared with the other groups, being exceptionally rare in 129V-type 2, 129V-type 1, and 129M-type “i” groups (9.1%, 11.1%, and 0%, respectively) (Supplementary Table 11).

Supplementary Table 1. Summary of CJD cases selected for biochemical analyses.

**gCJD/FFI**	**sCJD**	**PK titration (gCJD/sCJD analyzed)**	**TSA (gCJD/sCJD analyzed)**
**129M-type 1**	**MM(V)1**	**15/6**	**15/6**
E200K		8	8
4/5OPRI		3	3
V210I		4	4
**129V-type 2**	**VV2, MV2K**	**12/7, 5**	**12/13, 10**
5OPRI		6	6
E200K		3	3
E196K		1	1
T188A		1	1
R208H		1	1
**129V-type 1**	**VV1**	**8/7**	**8/7**
D178N		5	5
T188R		3	3
**129M-type 2C** (E200K)	**MM2C**	**3/5**	**3/5**
**FFI** (D178N-129M)	**MM2T**	**6/6**	**6/6**
**129M-type "i"** (E200K)		**6**	**6**
**Total**		**50/36**	**50/47**

List of abbreviations: gCJD, genetic Creutzfeldt-Jakob disease; FFI, fatal familial insomnia; sCJD, sporadic CJD; PK, proteinase K; TSA, thermosolubility assay.

Supplementary Table 2. *PRNP* genotype and PrP^{Sc} type in 200 unselected sCJD cases.

		Codon 129 polymorphism			
		**MM**	**MV**	**VV**	**Total**
n		129	31	40	200
**PrP^{Sc} type**	**1**	67 (51.9)	2 (6.5)	2 (5.0)	71 (35.5)
	**1+2**	56 (43.4)	7 (22.6)	6 (15.0)	69 (34.5)
	**2**	6 (4.6)	22 (70.9)	32 (80.0)	60 (30.0)

Adapted from [7].

Supplementary Table 3. Co-occurrence of PrP^{Sc} type 1+2 in the cases with at least six brain regions analyzed and regional distribution of mixed PrP^{Sc} types.

**Codon 129**	**129M**									**129V**					
***PRNP* mutation**	**D178N**	**T196A/K**	**E200K**	**V203I**	**R208H**	**V210I**	**3/4-OPRI**	**5/6-OPRI**	**Others A[a]**	**D178N**	**T188R**	**E200K**	**5/6-OPRI**	**Others B[b]**	**Total (%)**
n	13	6	45	3	5	43	3	1	4	3	6	3	5	2	142
**Overall**	0	1 (16.7)	0	1 (33.3)	0	4 (9.3)	0	0	0	0	1 (16.7)	0	3 (60.0)	0	10 (7.1)
**FC**	-	1 (100)	-	0	-	0	-	-	-	-	0	-	3 (100)	-	4 (40.0)
**TC**	-	1 (100)	-	1 (100)	-	3 (75.0)	-	-	-	-	0	-	1 (33.3)	-	6 (60.0)
**OC**	-	1 (100)	-	0	-	2 (50.0)	-	-	-	-	0	-	2 (66.7)	-	5 (50.0)
**PUT**	**-**	1 (100)	**-**	0	**-**	2 (50.0)	**-**	**-**	**-**	**-**	1 (100)	**-**	2 (66.7)	**-**	6 (60.0)
**TH**	**-**	1 (100)	**-**	1 (100)	**-**	3 (75.0)	**-**	**-**	**-**	**-**	1 (100)	**-**	3 (100)	**-**	9 (90.0)
**CE**	**-**	0	**-**	0	**-**	1 (25.0)	**-**	**-**	**-**	**-**	1 (100)	**-**	3 (100)	**-**	5 (50.0)

[a]Others A included: R148H (*n* = 1), T188K (*n* = 1), E211D (*n* = 1), and T183A (*n* = 1).

[b]Others B included: T188A (*n* = 1), and R208H (*n* = 1).

List of abbreviations: FC, frontal cortex; TC, temporal cortex; OC, occipital cortex; PUT, putamen; TH, thalamus; CE, cerebellum.

Supplementary Table 4. Glycoform ratio across the spectrum of gCJD variants and mutations, and comparison with sCJD subtypes.

Group	*n*	diglycosylated (%)	monoglycosylated (%)	unglycosylated (%)
**gCJD 129M-type 1**				
E200K	9	51.0±3.6	36.0±2.2	12.0±2.2
4 to 6-OPRI	7	28.9±5.4	46.2±2.8	24.9±5.0
V210I	9	32.7±2.3	45.9±2.4	21.4±3.5
**sCJD MM(V)1**	22	32.0±2.6	43.7±3.1	24.3±3.7
**gCJD 129V-type 2**				
E200K	4	57.9±11.7	34.9±7.7	7.2±3.9
5/6-OPRI	4	36.4±3.5	47.8±3.6	15.8±4.2
**sCJD VV2**	15	36.8±3.1	42.1±1.5	21.1±3.5
**sCJD MV2K**	7	30.2±3.2	42.2±2.4	27.2±4.0
**gCJD 129V-type 1**				
D178N	9	46.5±5.6	40.6±1.6	12.9±4.6
T188R	5	26.8±3.2	43.5±3.2	29.7±5.3
**sCJD VV1**	5	23.0±2.7	46.4±2.1	30.6±1.4
**gCJD 129M-type 2C**				
E200K	3	53.4±2.8	36.5±1.1	10.1±3.4
**sCJD MM2C**	6	31.2±2.2	42.4±2.9	26.4±3.2
**FFI (D178N-129M)**	6	60.9±7.5	34.2±6.4	4.9±3.8
**sCJD MM2T**	5	33.2±2.7	42.5±2.1	24.3±1.2
**gCJD 129M-type "i"**				
E200K	7	50.3±5.6	39.5±4.2	10.2±1.7
**Atypical gCJD**				
T183A	2	18.9±1.1	71.8±2.5	9.3±1.4
5/6-OPRI	3	2.9±5.0	50.9±7.2	46.2±9.3

Supplementary Table 5. Patterns of PrP deposition across the spectrum of sCJD phenotypes.

sCJD phenotype	*n*	Cerebellar or cortical synaptic (%)	Cortical perivacuolar/coarse (%)	Cerebellar plaque-like deposits (%)	Cerebellar kuru plaques (%)
**MM(V)1**	120	120 (100)	57 (47.5)	0	0
**VV1**	5	5 (100)	0	0	0
**MM2C**	13	8 (61.5)	13 (100)	0	0
**MM2T**	6	5 (83.3)	0	0	0
**MV2K**	23	23 (100)	4 (17.4)	23 (100)	23 (100)
**VV2**	39	39 (100)	0	39 (100)	0

Mixed sCJD types were merged with the corresponding "pure" subtype based on similarities of the predominant histo-molecular phenotype. Accordingly, the pairs MM(V)1/MM(V)1+2C were included into the MM(V)1) group, MM2C/MM2C+1 into the MM2C group, MV2K/MV2K+2C into the MV2Kgroup, and VV2/VV2+1 into the VV2 group. Adapted from [7].

Supplementary Table 6. Comparison of age at onset and disease duration between 129 homozygotes (MM, VV) and 129 heterozygotes (MV, VM) according to genetic groups and *PRNP* mutations.

		**Age at onset (yrs.)**			**Disease duration (mo.)**		
	n	**129 homozygous**	**129 heterozygous**	***p***	**129 homozygous**	**129 heterozygous**	***p***
**129M-type 1**	**119/22**	**63.5±9.9**	**62.6±12.6**	**ns**	**6.5±14.1**	**11.1±26.6**	**ns**
E200K	53/5	61.7±8.3	66.0±18.1	ns	4.5±2.7	4.4±3.2	ns
V210I	40/11	65.6±10.1	60.9±7.0	ns	3.9±3.4	3.1±2.5	ns
3/4-OPRI	6/0	65.0±3.9	-	-	8.6±4.6	-	ns
5/6-OPRI	4/2	46.0±8.6	45.5±9.2	ns	56.5±60.9	84.0±50.9	ns
Others A[a]	16/4	67.6±11.2	71.5±12.7	ns	6.2±6.7	5.2±5.3	ns
**129V-type 2**	**12/5**	**63.7±4.5**	**63.0±14.7**	**ns**	**8.2±7.5**	**10.6±4.6**	**ns**
E200K	3/2	59.7±6.0	57.5±13.4	ns	5.3±4.0	9.5±2.1	ns
5/6-OPRI	6/3	64.2±2.8	66.7±17.0	ns	6.4±3.2	11.3±6.1	ns
Others B[¶]	3/0	66.7±4.0	-	-	17.0±15.56	-	-
**129V-type 1**	**9/8**	**50.1±13.4**	**52.5±9.1**	**ns**	**14.3±4.7**	**20.9±12.6**	**ns**
D178N	5/7	40.4±4.5	49.6±3.9	0.0088	17.2±3.7	22.3±12.9	ns
T188R	4/1	62.2±10.0	73.0	**-**	10.7±3.2	11.0	-
**129M-type 2 FFI**							
D178N	**7/6**	**57.5±6.5**	**50.3±10.1**	**ns**	**9.1±1.9**	**26.2±3.9**	**<0.0001**
**129M-type 2C**	**1/4**	**57**	**63.7±7.4**	-	**1.5**	**37.7±39.0**	-
E200K	1/3	57	63.3±9.1	-	1.5	18.3±4.6	-
5-OPRI	0/1	-	65	-	-	96	-

129M-type “i” and atypical groups have been omitted because of the former included only codon 129 heterozygous (MV) subjects, whereas the latter only codon 129 homozygous (either MM or VV).

[a]Others A included: R208H, *n* = 7; V203I, n = 3; E196A/K, *n* = 6; T188K, *n* = 1; R148H, *n* = 1; D211Q, *n* = 2.

[b]Others B included: R208H, *n* = 1; E196K, *n* = 1; T188A, *n* = 1.

Supplementary Table 7. Symptoms and signs at disease onset in gCJD groups and *PRNP* mutations.

**Diagnostic group**	**129M-type 1**				**129V-type 2**			**129V-type 1**			**129M-type 2C**	**129M-type "i"**	**Atypical**
**Mutation**	**All**	**E200K**	**V210I**	**OPRI**	**All**	**OPRI**	**E200K**	**All**	**D178N**	**T188R**	**All**	**All**	**All**[a]
n	138	57	51	12	14	7	4	11	8	3	4	7	5
Cognitive[b]	79 (57.2)	32 (56.1)	27 (52.9)	7 (58.3)	6 (42.9)	4 (57.1)	1 (25.0)	9 (81.8)	7 (87.5)	2 (66.6)	3 (75.0)	4 (57.1)	3 (60.0)
Visual[c]	25 (18.1)	6[#] (10.5)	11 (21.6)	0	0	0	0	0	0	0	0	1 (14.3)	0
Ataxia/ cerebellar	69 (50.0)	32 (56.1)	22 (43.1)	4 (33.3)	11 (78.6)	5 (71.4)	4 (100)	1 (9.1)	0	1 (33.3)	0	5 (71.4)	3 (60.0)
Myoclonus	3 (2.2)	1 (1.8)	2 (3.9)	0	0	0	0	0	0	0	0	0	0
Other dyskinesia(s)	9 (6.5)	2 (3.5)	7 (13.7)	0	0	0	0	0	0	0	0	0	0
Parkinsonism	9 (6.5)	4 (7.0)	3 (5.9)	0	2 (14.3)	2 (28.6)	0	2 (18.2)	2 (25.0)	0	0	0	1 (20.0)
Pyramidal	6 (4.3)	3 (5.3)	3 (5.9)	1 (8.3)	0	0	0	0	0	0	0	0	0
Sensory	11 (8.0)	6 (10.5)	4 (7.8)	2 (16.6)	0	0	0	0	0	0	0	1 (14.3)	1 (20.0)
Behavioral/ psychiatric[d]	25 (18.1)	11 (19.3)	9 (17.6)	3 (25.0)	2 (14.3)	2 (28.6)	0	2 (18.2)	1 (12.5)	1 (33.3)	2 (50.0)	1 (14.3)	0
Unilateral	23 (16.7)	12 (21.1)	11 (21.6)	1 (8.3)	0	0	0	0	0	0	0	0	0

[a]Included T183A-129M/PrP^{Sc} type 2 (n=2) and 5/6-OPRI-129V/PrP^{Sc} type 1+2 (n=3).

[b]One or more of: memory loss, aphasia, confusion and/or disorientation, intellectual decline.

[c]One or more of: visual loss, visual field defect, visual distortion, abnormal color vision, cortical blindness.

[d]One or more of: depression or anxiety of recent onset requiring psychiatric evaluation, delusions, hallucinations, panic attacks, psychosis, and behavioral changes.

[#]Statistic significant comparisons between *PRPN* mutations within each genetic group: visual E200K vs. V210I, $p \leq 0.05$ (group 129M-type 1). No significant differences were detected between carriers of the same *PRNP* mutation belonging to different groups.

Supplementary Table 8. Symptoms and signs during the entire disease course across the spectrum of gCJD groups.

**Diagnostic group**	**129M-type 1**				**129V-type 2**			**129V-type 1**			**129M-type 2C**	**129M-type "i"**	**Atypical**
**Mutation**	**All**	**E200K**	**V210I**	**OPRI**	**All**	**OPRI**	**E200K**	**All**	**D178N**	**T188R**	**All**	**All**	**All**[a]
n	138	57	51	12	14	7	4	12	8	4	4	7	5
Cognitive[b]	132 (95.7)	56 (98.2)	46 (90.2)	12 (100)	13 (92.9)	7 (100)	3 (75.0)	11 (91.7)	7 (87.5)	4 (100)	4 (100)	7 (100)	5 (100)
Visual[c]	40 (29.0)	17 (29.8)	17 (33.3)	3 (25.0)	0	0	0	1 (8.3)	1 (12.5)	0	0	2 (28.6)	0
Ataxia/ cerebellar	114 (82.6)	46 (80.7)	40 (78.4)	12 (100)	14 (100)	7 (100)	4 (100)	5 (41.7)	2 (25.0)	3 (75.0)	2 (50.0)	6 (85.7)	4 (80.0)
Myoclonus	101 (73.2)	44 (77.2)	34 (66.7)	11 (91.7)	4 (21.4)	2 (28.6)	0	7 (58.3)	7 (87.5)	0	2 (50.0)	1 (14.3)	2 (40.0)
Other dyskinesia(s)	31 (22.5)	10 (17.5)	17 (33.3)	2 (16.7)	1 (7.1)	0	0	4 (33.3)	3 (.37.5)	1 (25.0)	0	0	0
Parkinsonism	38 (27.5)	17 (29.8)	13 (25.5)	5 (41.7)	6 (42.9)	4 (57.1)	0	5 (41.7)	5 (62.5)	0	1 (25.0)	2 (28.6)	3 (60.0)
Pyramidal	73 (52.9)	34 (59.6)	25 (49.0)	7 (58.3)	8 (57.1)	3 (42.9)	2 (50.0)	4 (33.3)	4 (50.0)	0	1 (25.0)	2 (28.6)	2 (40.0)
Sensory	22 (15.9)	9 (15.8)	10 (19.6)	2 (16.7)	0	0	0	0	0	0	0	2 (28.6)	1 (20.0)
Behavioral/ psychiatric[d]	51 (37.0)	20 (35.1)	19 (37.3)	5 (41.7)	8 57.1)	4 (57.1)	2 (50.0)	4 (33.3)	2 (25.0)	2 (50.0)	3 (75.0)	6 (85.7)	2 (40.0)

[a]Included T183A-129M/PrP^{Sc} type 2 (n=2) and 5/6-OPRI-129V/PrP^{Sc} type 1+2 (n=3).

[b]One or more of: memory loss, aphasia, confusion and/or disorientation, intellectual decline.

[c]One or more of: visual loss, visual field defect, visual distortion, abnormal color vision, cortical blindness.

[d]One or more of: depression or anxiety of recent onset requiring psychiatric evaluation, delusions, hallucinations, panic attacks, psychosis, and behavioral changes.

Supplementary Table 9. Results of CSF analyses in gCJD groups.

Diagnostic group	14-3-3 protein positive/tested (%)	t-tau >1250 pg/ml positive/tested (%)	prion RT-QuIC positive/tested (%)		
			Full-length PrPrec (Ha23-231)	Truncated PrPrec (Ha90-231)	Overall
**129M-type 1**	**73/87 (83.9)**	**28/29 (96.5)**	**24/24 (100)**	**13/13 (100)**	**27/27 (100)**
E200K	20/26 (76.9)	9/9 (100)	8/8 (100)	5/5 (100)	8/8 (100)
V210I	29/37 (78.4)	11/11 (100)	8/8 (100)	6/6 (100)	10/10 (100)
3 to 6-OPRI	7/7 (100)	2/3 (66.7)	2/2 (100)	1/1 (100)	2/2 (100)
Others A[a]	17/17 (100)	6/6 (100)	6/6 (100)	1/1 (100)	7/7 (100)
**129V-type 2**	**9/10 (90.0)**	**4/5 (80.0)**	**4/5 (80.0)**	**1/1 (100)**	**4/5 (80.0)**
E200K	3/3 (100)	1/2 (50.0)	2/2 (100)	1/1 (100)	2/2 (100)
5/6-OPRI	3/4 (75.0)	2/2 (100)	2/2 (100)	-	2/2 (100)
Others B[b]	3/3 (100)	1/1 (100)	0/1 (0.0)	-	0/1 (0.0)
**129V-type 1**	**3/5 (60.0)**	**5/5 (100)**	**1/3 (33.3)**	-	**1/3 (33.3)**
D178N	2/2 (100)	2/2 (100)	0/1 (0.0)	-	0/1 (0.0)
T188R	1/3 (33.3)	3/3 (100)	1/2 (50.0)	-	1/2 (0.0)
**129M-type 2C** (E200K)	**3/3 (100)**	**3/3 (100)**	**1/1 (100)**	**-**	**1/1 (100)**
**129M-type “i” (**E200K)	**5/5 (100)**	**1/3 (33.3)**	**2/2 (100)**	**1/1 (100)**	**2/2 (100)**
**Atypical** (5/6-OPRI)	**2/3 (66.7)**	**0/1 (0.0)**	**-**	**-**	**-**
**Total**	**95/113 (84.1)**	**41/46 (89.1)**	**32/35 (91.4)**	**15/15 (100)**	**35/38 (92.1)**

[a]Others A included: R208H, *n* = 6; V203I, *n* = 3; E196A/K, *n* = 4; R148H, *n* = 1; T188K, *n* = 1; E211Q, *n* = 2.

[b]Others B included: T188A, *n* = 1; E196K, *n* = 1; R208H, *n* = 1.

Supplementary Table 10. Brain MRI findings in gCJD groups.

Diagnostic group	n	Typical (% of tested)	Distribution of abnormal signal		Timing (mo.)
			CTX (% of positive)	STR (% of positive)	
**129M-type 1**	**51**	**45 (88.2)**	**21 (46.6)**	**40 (88.8)**	**2.3±2.0**
E200K	18	17 (94.4)	8 (47.1)	17 (100)	1.9±1.6
V210I	20	19 (95.0)	7 (36.8)	16 (84.2)	1.9±1.2
4/5-OPRI	2	1 (50.0)	-	1 (100)	8.3±3.9
Others A[a]	11	8 (72.7)	6 (75.0)	6 (75.0)	2.4±1.9
**129V-type 2**	**9**	**5 (55.6)**	**0 (0.0)**	**5 (100)**	**6.0±7.6**
E200K	4	2 (50.0)	-	2 (100)	3.3±0.6
5/6-OPRI	3	2 (66.7)	-	2 (100)	2.0±1.4
Others B[b]	2	1 (50.0)	-	1 (100)	14.0±12.7
**129V-type 1**	**3**	**2 (66.7)**	**2 (100)**	**0 (0.0)**	**6.7±1.4**
D178N	1	1 (100)	1 (100)	-	**6**
T188R	2	1 (50.0)	1 (100)	-	7.0±1.4
**129M-type 2C** (E200K)	**2**	**2 (100)**	**2 (100)**	**1 (50.0)**	**9.5±12.0**
**129M-type "i"** (E200K)	**2**	**1 (50.0)**	**0 (0.0)**	**1 (100)**	**6.5±4.9**
**Total**	**67**	**55 (82.1)**	**25 (45.5)**	**47 (85.4)**	**3.3±4.0**

According to current diagnostic criteria for sCJD [4], brain MRI was defined "typical" in the presence of hyperintensities on T2-FLAIR and/or DWI sequences in at least two cortical regions (temporal, parietal and occipital cortices) and/or the striatum.

[a]Others A included: R208H, *n* = 4; E196K/A, *n* = 5; T188K, *n* = 1; E211Q, *n* = 1.

[b]Others B included: T188A, *n* = 1; E196K, *n* = 1.

List of abbreviations: CTX: neocortices; STR: striatum.

Supplementary Table 11. Electroencephalographic findings in gCJD groups.

		EEG activity			
**Diagnostic group**	**n**	**PSWCs (%)**	**Paroxysmal (%)**	**Slowing (%)**	**Normal (%)**
**129M-type 1**	**132**	**83 (62.9)**	**15 (11.4)**	**34 (25.7)**	**0 (0.0)**
E200K	55	34 (61.8)	6 (10.9)	15 (27.3)	-
V210I	48	31 (64.6)	7 (14.6)	10 (20.8)	-
3 to 6OPRI	11	5 (45.5)	1 (9.0)	5 (45.5)	-
Others A[a]	18	13 (72.2)	1 (5.5)	4 (22.2)	-
**129V-type 2**	**11**	**1 (9.1)**	**1 (9.1)**	**9 (81.8)**	**0 (0.0)**
E200K	4	-	-	4 (100)	-
5/6-OPRI	5	1 (20.0)	1 (20.0)	3 (60.0)	-
Others B[b]	2	-	-	2 (100)	-
**129V-type 1**	**9**	**1 (11.1)**	**1 (11.1)**	**6 (66.7)**	**1 (11.1)**
D178N	6	1 (16.7)	1 (16.7)	3 (50.0)	1 (16.7)
T188R	3	-	-	3 (100)	-
**129M-type 2C**	**5**	**2 (40.0)**	**0 (0.0)**	**2 (40.0)**	**1 (20.0)**
E200K	4	2 (50.0)	-	1 (25.0)	1 (25.0)
5-OPRI	1	-	-	1 (100)	-
**129M-type "i"** (E200K)	**6**	**0 (0.0)**	**0 (0.0)**	**6 (100)**	**0 (0.0)**
**Atypical**	**4**	**2 (50.0)**	**0 (0.0)**	**0 (0.0)**	**2 (50.0)**
T183A	2	-	-	-	2 (100)
5/6-OPRI	2	2 (100)	-	-	-
**Total**	**167**	**89 (53.3)**	**17 (10.2)**	**57 (34.1)**	**4 (2.4)**
**Time of appearance (mo.)**		**2.8±2.3**	-	-	-

[a]Others A included: R208H, *n* = 7; V203I, *n* = 2; E196A/K, *n* = 5; T188K, *n* = 1; R148H, *n* = 1; D211Q, *n* = 2.

[b]Others B included: E196K, *n* = 1; R208H, *n* = 1.

List of abbreviations: PSWCs: Periodic Sharp Wave Complexes.

Supplementary Fig. 1 Immunoblot profile of PrP^{Sc} core fragments in two patients with "atypical" 5/6-OPRI-129V.

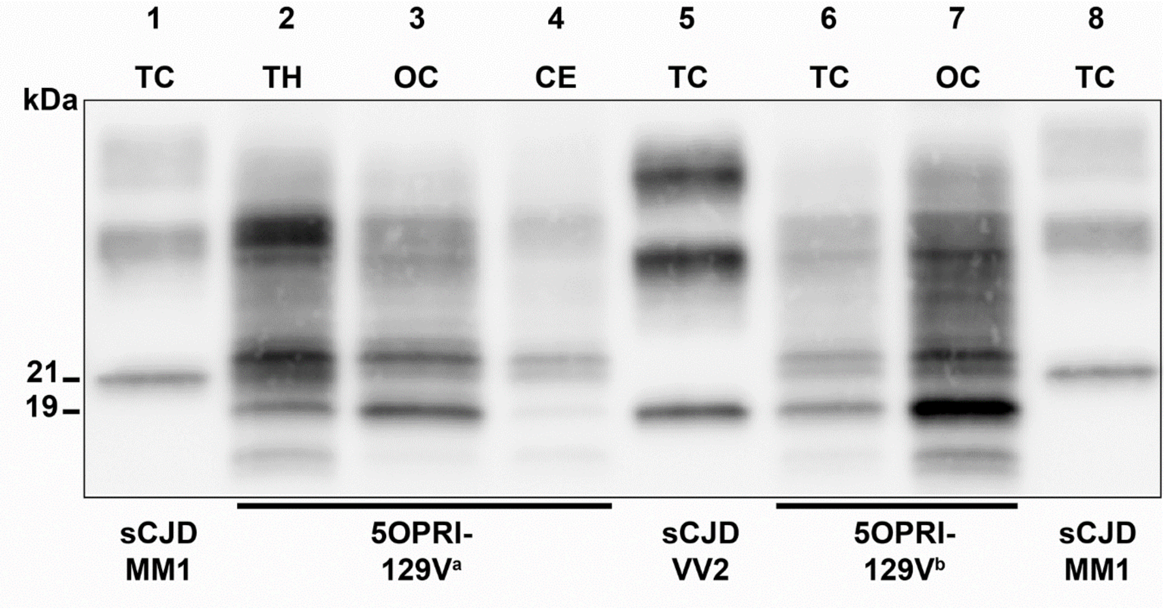

[a]Patient a (lanes 2-4), [b]patient b (lanes 6 and 7). Both cases showed the co-occurrence of PrP^{Sc} types 1 and 2. However, the PrP^{Sc} "type 1" profile comprised a doublet of fragments, including a second atypical fragment migrating slightly slower than the typical 21 kDa associated band. An additional band migrating at ~17 kDa was evident in both patients. The immunoblot was probed with the primary antibody 3F4.

List of abbreviations: TC, temporal cortex; TH, thalamus; OC, occipital cortex.

Supplementary Fig. 2 Immunoblot profile of PrP^{Sc} core fragments in multiple brain regions in a subject carrying the T183A-129M haplotype.

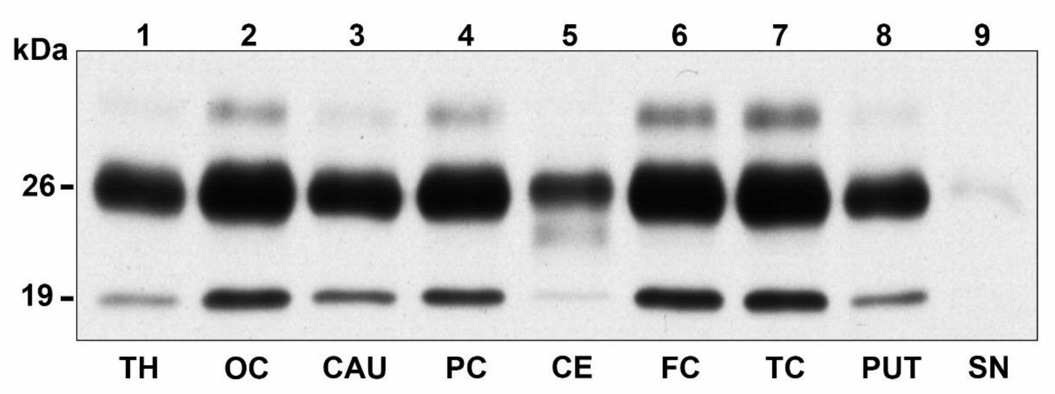

PrP^{Sc} was detected in all the brain regions analyzed, although significantly lower amounts of protein were found in the cerebellum and midbrain. The immunoblot profile of PrP^{Sc} was characterized by predominance of the monoglycosylated band and marked underrepresentation of the diglycosylated isoform.

List of abbreviations: TH, thalamus; OC, occipital cortex; CAU, caudate nucleus; PC, parietal cortex; CE, cerebellum; FC, frontal cortex; TC, temporal cortex; PUT, putamen; SN, substantia nigra.

Supplementary Fig. 3 Lesion profiles according to codon 129 genotype in each haplotype/PrP^{Sc} type group.

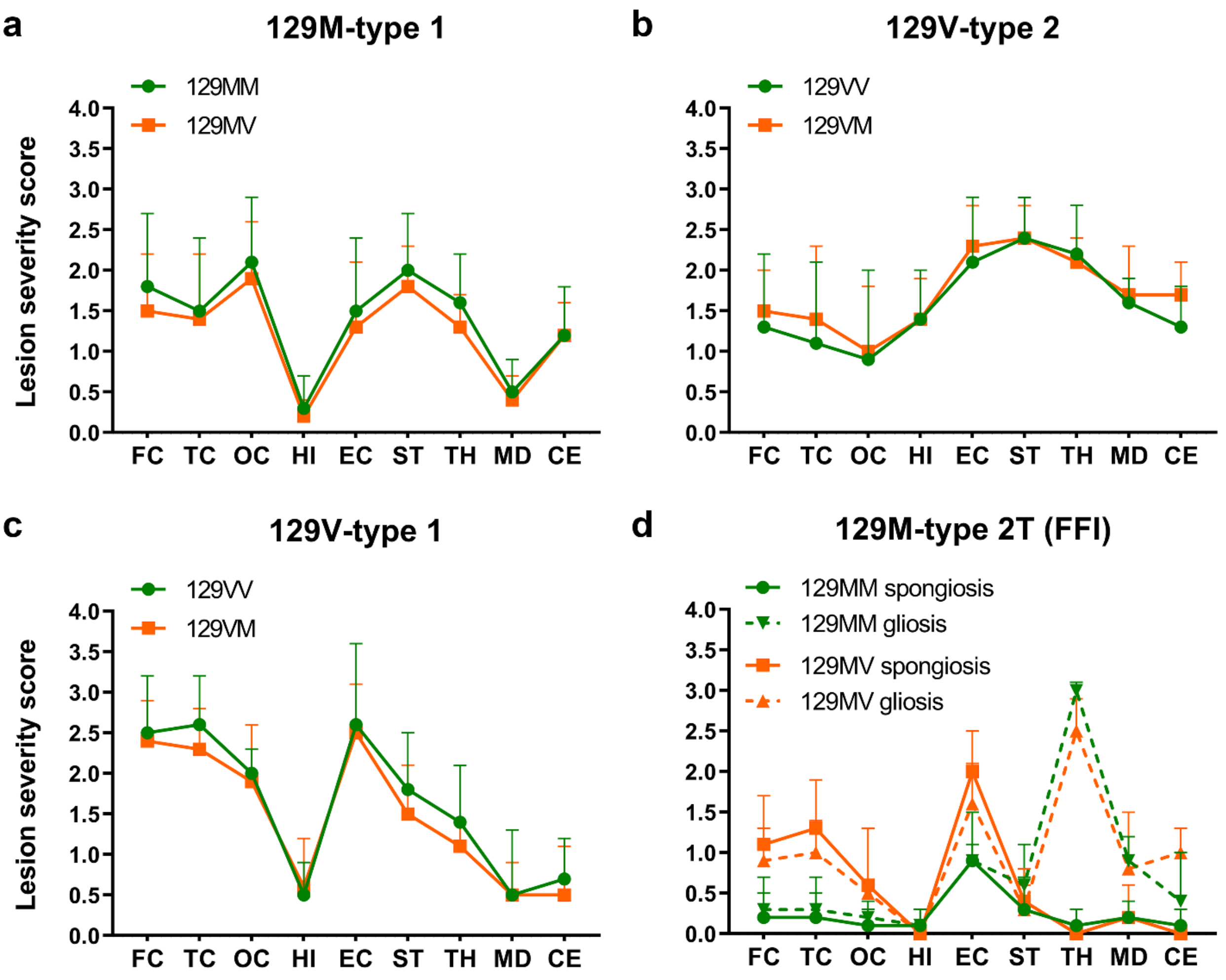

Lesion profiles were obtained by averaging the scores of spongiform change and astrogliosis for each brain region examined (the two scores are shown separately in FFI). Data are expressed as mean ± SD values Groups including less than 5 homozygous or heterozygous patients are not shown.

List of abbreviations: FC, frontal cortex; TC, temporal cortex; OC, occipital cortex; HI, hippocampus; EC, entorhinal cortex; ST, neostriatum; TH, thalamus; MD, midbrain; CE, cerebellum.

Supplementary Fig. 4 Evaluation of aggregate diameter in 129V-type 1 and type 2 cases and comparison with sCJD VV1 and VV2 subtypes.

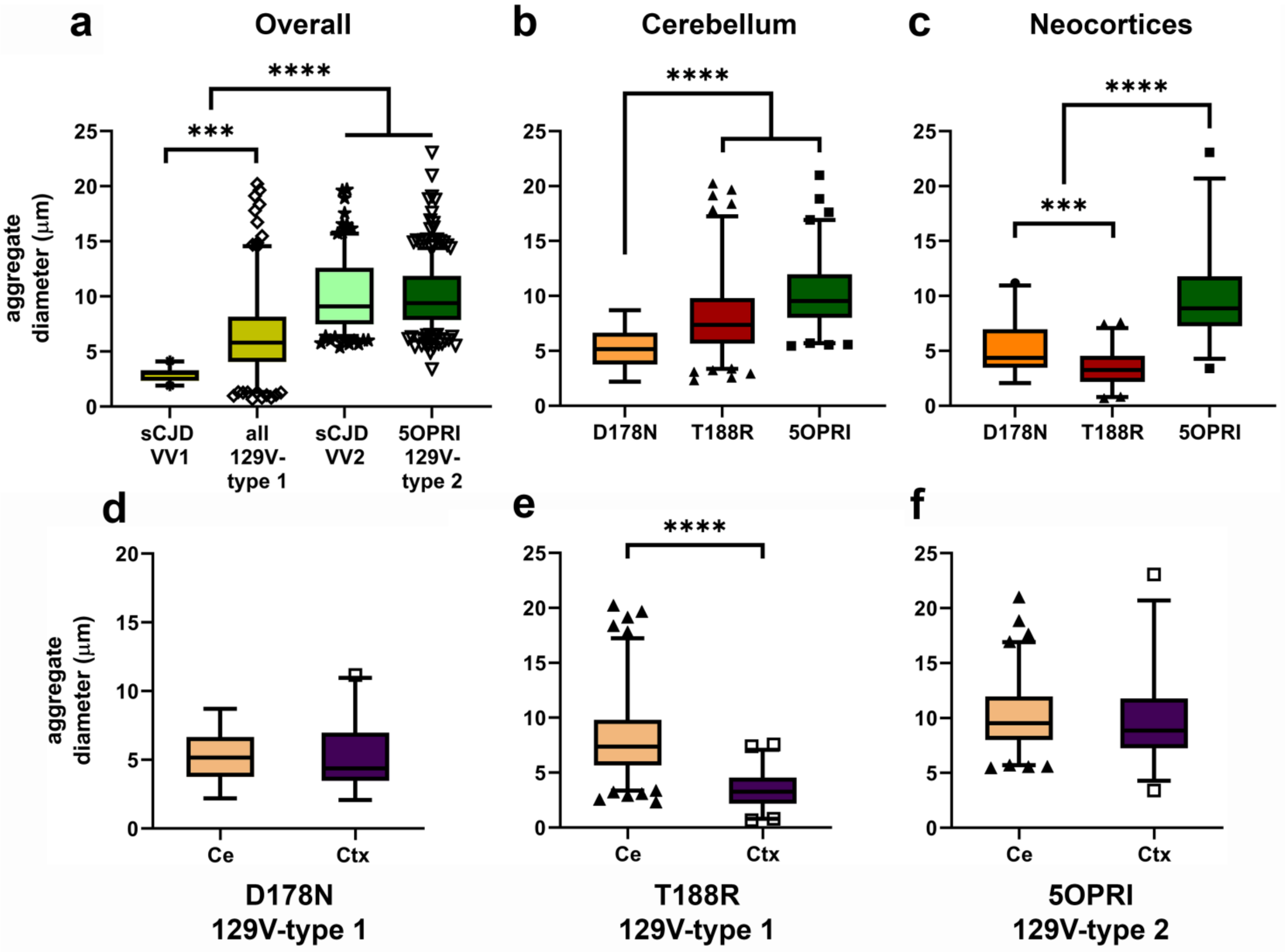

Overall, the 129V-type 2 combination showed PrP^{Sc} plaque-like aggregates of larger diameter than 129V-type 1 (a); aggregate diameter was consistent between genetic 129V-type 2 and sCJD VV2, whereas in sCJD VV1 PrP^{Sc} deposits were significantly smaller than in genetic 129V-type 1 (a). Within the latter group, subjects carrying D178N displayed smaller PrP^{Sc} aggregates than those with T188R in the cerebellum (b) and, on the contrary, larger in the neocortices (c). The finding mainly depends on the heterogeneity of PrP^{Sc} aggregate size between cerebellum and neocortex in T188R cases (e); in contrast, the aggregate size did not differ significantly between the two brain regions in the D178N (d) and 5OPRI-129V type 2 cases (f).

Legend: **** $p \leq 0.0001$, *** $p \leq 0.001$.

Supplementary Fig. 5 Lesion profiles according to *PRNP* mutations in each haplotype/PrP^{Sc} type combination.

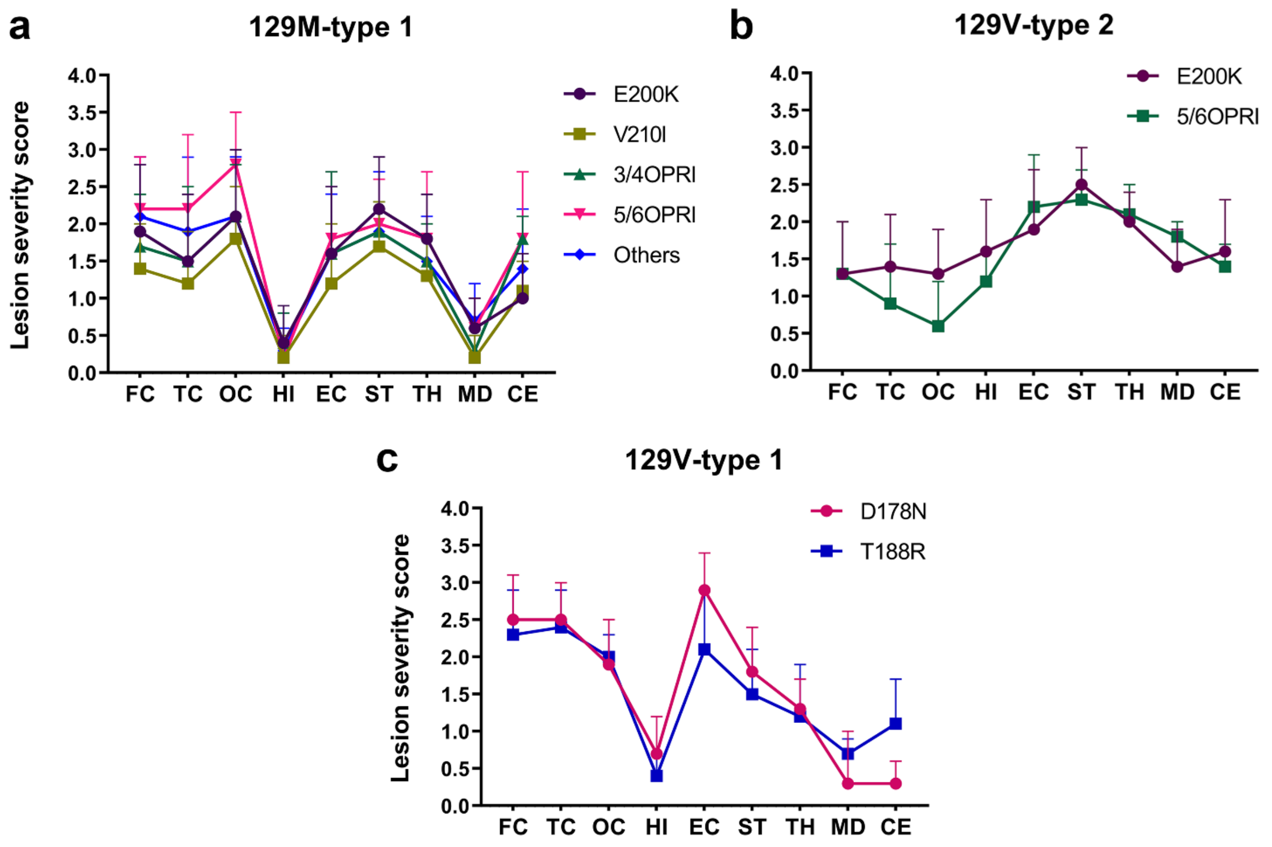

Lesion profiles were obtained by averaging the scores of spongiosis and gliosis for each brain region examined. Data are expressed as mean ± SD values. Only mutations occurring in at least 5 patients are included. Groups including a single *PRNP* mutation are not shown.

List of abbreviations: FC, frontal cortex; TC, temporal cortex; OC, occipital cortex; HI, hippocampus; EC, entorhinal cortex; ST, neostriatum; TH, thalamus; MD, midbrain; CE, cerebellum.

Supplementary Fig. 6 Lesion profiles of T183A-129M and “atypical” 129V-5/6-OPRI.

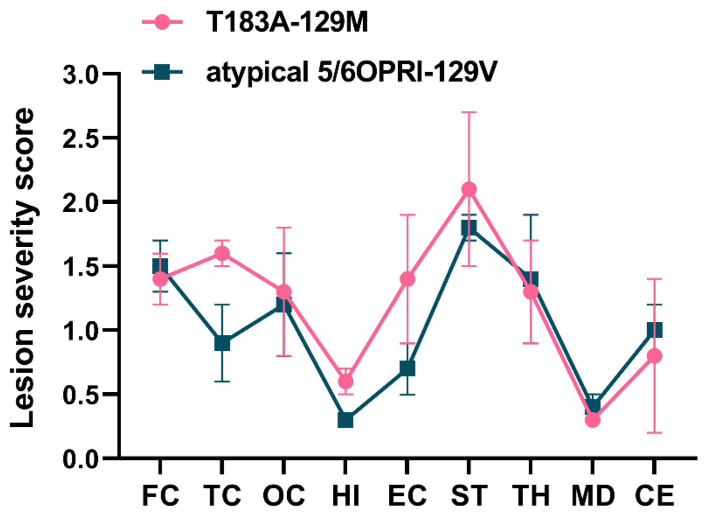

Lesion profiles were obtained by averaging the scores of spongiosis and gliosis for each brain region examined. Data are expressed as mean ± SD values.

List of abbreviations: FC, frontal cortex; TC, temporal cortex; OC, occipital cortex; HI, hippocampus; EC, entorhinal cortex; ST, neostriatum; TH, thalamus; MD, midbrain; CE, cerebellum.

Supplementary Fig. 7 Comparison of PrP^{Sc} PK resistance (a-c) and "thermosolubility" (d-f) among *PRNP* mutations in each haplotype/PrP^{Sc} type group.

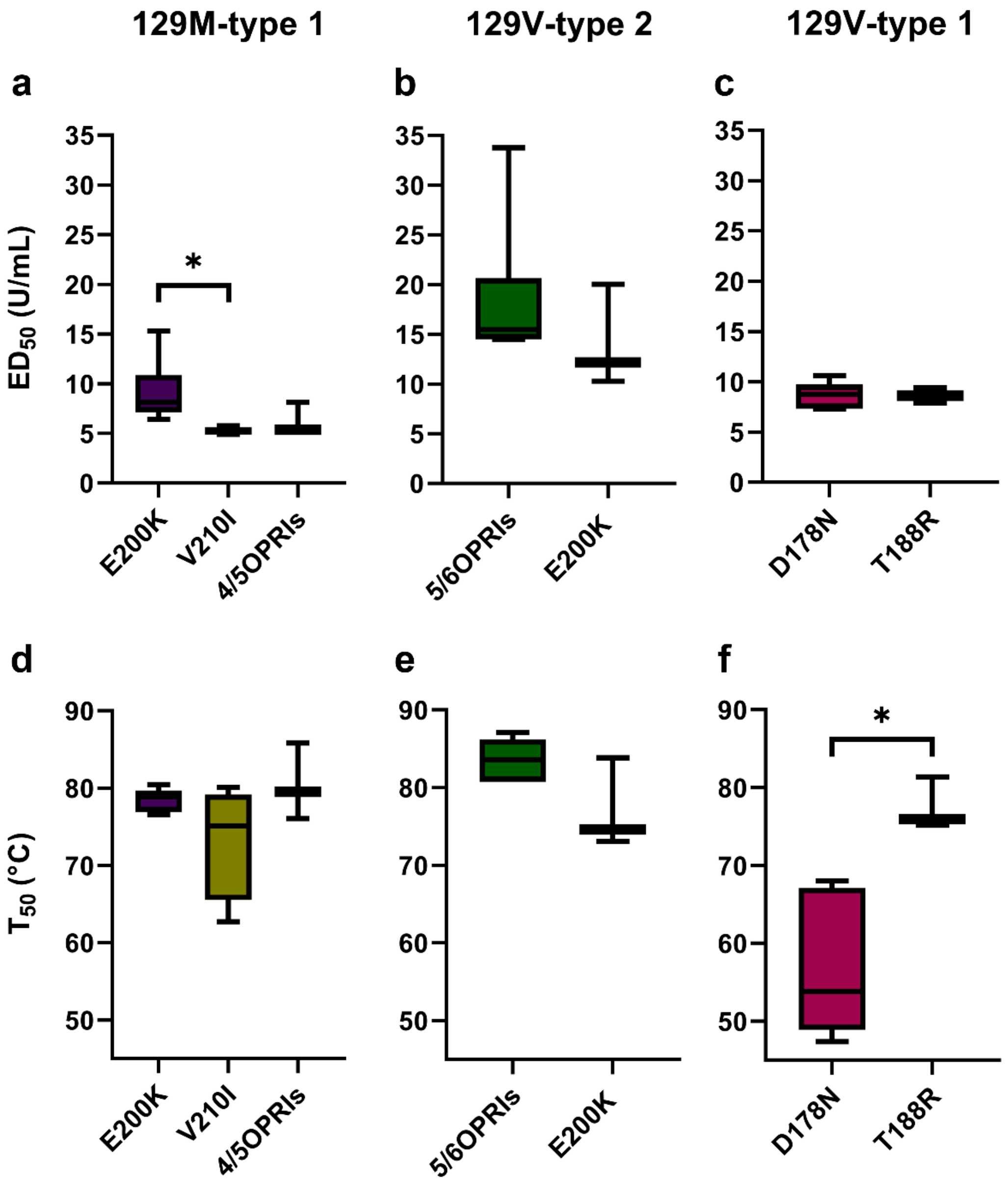

No significant differences in ED_{50} were detected among *PRNP* variants in gCJD, except for a slightly higher PrP^{Sc} PK resistance in E200K compared to V210I in the 129M-type1 group (a). Similarly, no significant differences in T_{50} were noted, except for a higher PrP^{Sc} sensitivity to temperature denaturation in D178N than T188R in the 129V-type 1 group (f). Only mutations occurring in at least 3 patients are included. Groups including a single *PRNP* mutation are not shown.

Legend: * p≤0.05

Supplementary references

[1] Brown P, Gibbs CJ Jr, Rodgers-Johnson P, et al. (1994) Human spongiform encephalopathy: the National Institutes of Health series of 300 cases of experimentally transmitted disease. Ann Neurol 35:513-529

[2] Foutz A, Appleby BS, Hamlin C, et al. (2017) Diagnostic and prognostic value of human prion detection in cerebrospinal fluid. Ann Neurol 81:79-92

[3] Franceschini A, Baiardi S, Hughson AG, et al. (2017) High diagnostic value of second generation CSF RT-QuIC across the wide spectrum of CJD prions. Sci Rep 7:10655.

[4] Hermann P, Appleby B, Brandel JP, et al. (2021) Biomarkers and diagnostic guidelines for sporadic Creutzfeldt-Jakob disease. Lancet Neurol 2021 20:235-246

[5] McGuire LI, Peden AH, Orrú CD, et al. (2012) Real time quaking-induced conversion analysis of cerebrospinal fluid in sporadic Creutzfeldt-Jakob disease. Ann Neurol 72:278-285

[6] Lattanzio F, Abu-Rumeileh S, Franceschini A, et al. (2017) Prion-specific and surrogate CSF biomarkers in Creutzfeldt-Jakob disease: diagnostic accuracy in relation to molecular subtypes and analysis of neuropathological correlates of p-tau and Aβ42 levels. Acta Neuropathol 133:559-578

[7] Parchi P, Strammiello R, Notari S, Giese A, Langeveld JP, Ladogana A, Zerr I, Roncaroli F, Cras P, Ghetti B, et al (2009) Incidence and spectrum of sporadic Creutzfeldt-Jakob disease variants with mixed phenotype and co-occurrence of PrPSc types: an updated classification. Acta Neuropathol 118:659-671

[8] Wieser HG, Schindler K, Zumsteg D (2006) EEG in Creutzfeldt-Jakob disease. Clin Neurophysiol 117:935-951
